# Genotype-Environment Interactions Reveal Causal Pathways That Mediate Genetic Effects on Phenotype

**DOI:** 10.1371/journal.pgen.1003803

**Published:** 2013-09-19

**Authors:** Julien Gagneur, Oliver Stegle, Chenchen Zhu, Petra Jakob, Manu M. Tekkedil, Raeka S. Aiyar, Ann-Kathrin Schuon, Dana Pe'er, Lars M. Steinmetz

**Affiliations:** 1 Gene Center, Ludwig-Maximilians-Universität München, Munich, Germany; 2 European Molecular Biology Laboratory, European Bioinformatics Institute, Wellcome Trust Genome Campus, Hinxton, Cambridge, United Kingdom; 3 European Molecular Biology Laboratory, Genome Biology Unit, Heidelberg, Germany; 4 Department of Biological Sciences, Columbia University, New York, New York, United States of America; 5 Stanford Genome Technology Center, Palo Alto, California, United States of America; Washington University School of Medicine, United States of America

## Abstract

Unraveling the molecular processes that lead from genotype to phenotype is crucial for the understanding and effective treatment of genetic diseases. Knowledge of the causative genetic defect most often does not enable treatment; therefore, causal intermediates between genotype and phenotype constitute valuable candidates for molecular intervention points that can be therapeutically targeted. Mapping genetic determinants of gene expression levels (also known as expression quantitative trait loci or eQTL studies) is frequently used for this purpose, yet distinguishing causation from correlation remains a significant challenge. Here, we address this challenge using extensive, multi-environment gene expression and fitness profiling of hundreds of genetically diverse yeast strains, in order to identify truly causal intermediate genes that condition fitness in a given environment. Using functional genomics assays, we show that the predictive power of eQTL studies for inferring causal intermediate genes is poor unless performed across multiple environments. Surprisingly, although the effects of genotype on fitness depended strongly on environment, causal intermediates could be most reliably predicted from genetic effects on expression present in all environments. Our results indicate a mechanism explaining this apparent paradox, whereby immediate molecular consequences of genetic variation are shared across environments, and environment-dependent phenotypic effects result from downstream integration of environmental signals. We developed a statistical model to predict causal intermediates that leverages this insight, yielding over 400 transcripts, for the majority of which we experimentally validated their role in conditioning fitness. Our findings have implications for the design and analysis of clinical omics studies aimed at discovering personalized targets for molecular intervention, suggesting that inferring causation in a single cellular context can benefit from molecular profiling in multiple contexts.

## Author Summary

A long-standing challenge in biology is to unravel the chain of molecular events linking genetic variation to phenotypes like disease. Identifying the genes that act as intermediates between the underlying genetic variation and the disease can offer new ways to intervene in its progression. While large-scale molecular profiles are an important starting point, it is difficult to distinguish causal relationships from correlative associations. In this study, our goal was to develop strategies to identify these causal intermediates. We studied the effects of genetic differences in baker's yeast on fitness in multiple environmental conditions. While genetic effects on fitness depended strongly on the environment, genetic effects on the expression of truly causal intermediate genes tended to persist despite environmental changes. This indicates that causal intermediates can be found among genes whose expression is affected by genetic variation independently of environment. We thus developed a statistical method to predict causal intermediates based on genetics, gene expression, and fitness in multiple environments. Our study has implications for the design and analysis of clinical molecular profiling efforts towards understanding how genetic variation causes disease, suggesting that multiple contexts (e.g., cell types) can be informative even if they are not afflicted by the disease.

## Introduction

**Figure 1:**
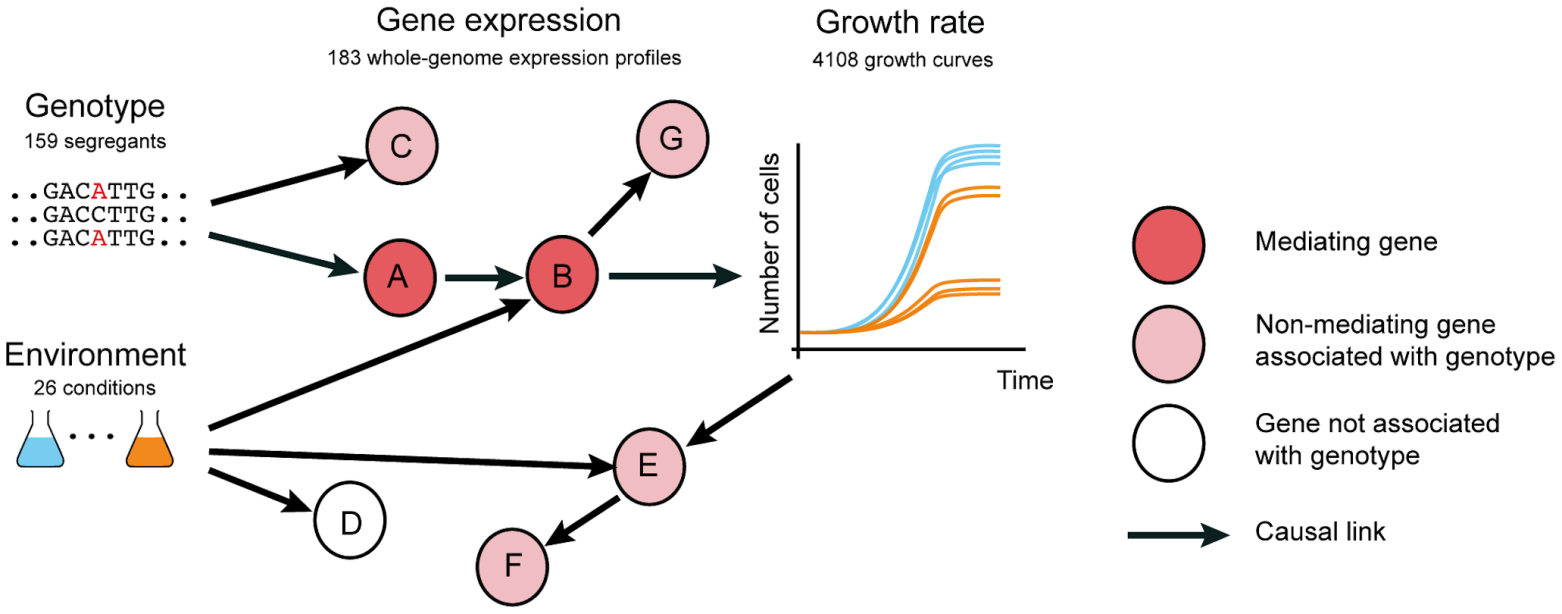
Distinguishing causal intermediate genes between genetic variation and phenotype. Genetic variants (genotype, left, here for 159 yeast segregants) affect physiological phenotype (growth curves, right) through a causal chain of molecular events (depicted as arrows) affecting expression of genes (nodes A and B). Genes like A and B that mediate the effect of genetic variation on phenotype are valuable molecular intervention points to counteract genetic defects that cause aberrant phenotypes. Genetic variants also affect expression of genes that are neither causal nor intermediates, including side effects (nodes C and G), and consequences of the physiological phenotype (nodes E and F). Thus, correlation between the expression of a gene and the genetic variant responsible for the phenotype is weak evidence that the expressed gene is a causal intermediate. Environment (colored flasks, left) causes (arrows) variation in gene expression and growth, yielding further perturbations that can be exploited to infer causal intermediates.

Genome-wide association studies have identified hundreds of genetic variants that increase susceptibility to diseases [1]. However, knowledge of the causal genetic variant, frequently occurring in genomic regions with little or no functional annotation, rarely yields opportunities for treatment. This may be because either the affected gene is not known, the affected gene is not druggable, or the pathway mediating the genetic effect on the physiological phenotype has not been elucidated. Therefore, the identification of the full intermediate pathways, including the causal intermediate molecules through which genetic variants affect physiological phenotypes (Fig. 1, nodes A and B), will greatly expand the set of possible targets for molecular intervention.

Transcription profiling has been utilized to narrow down the causal intermediate pathways and molecules between genotype and phenotypes of interest, by identifying genes whose expression levels are associated with genetic variants that also affect phenotype ([2], [3], [4] and reviewed in [5]). Using quantitative trait loci mapping, an abundance of genetic variants have been associated with gene expression levels (expression quantitative trait loci or eQTLs). In human, most eQTLs have been detected in the genomic vicinity of the associated gene, indicating a likely *cis* mechanism. However, as sample sizes increase, eQTLs that are located further away from the associated gene, presumably acting via a *trans* mechanism, are increasingly being detected [5]. Genes whose expression is associated with disease QTLs have been considered to be putative causal intermediates between genotype and the disease [5] (Fig. 1 node A, B). However, these genetic associations can also be the result of linkage (whereby the effect on gene expression is caused by a linked polymorphism, Fig. 1 node C) or reflect responses to the physiological phenotype (Fig. 1 nodes E, F). Hence, eQTL associations with genetic variants that also underlie phenotypes are merely correlative evidence, and alone do not confirm that the expression of these genes plays a causal role in phenotype. Thus far, a systematic evaluation to understand whether and under which circumstances eQTL association is predictive of causal intermediates has not been conducted.

It has long been known that genetic effects on physiological phenotypes often depend on the cellular and environmental context, such as culture media, cell type, or tissue. This dependency on cellular context, or genotype-environment interactions if the context is an external condition, has also been reported for genetic effects on gene expression, ranging from yeast to human [6], [7], [8]. Akin to the increased statistical power of multi-trait QTL mapping [9], detection of eQTLs is improved by mapping across multiple tissues [10]. However, the relevance of eQTLs detected in tissues that are not affected by the disease of interest for identifying causal intermediates remains unclear. Such investigations will be necessary to understand the utility of eQTL studies performed in proxy tissues [11].

Here we hypothesized that distinguishing causal intermediates from correlative associations can be facilitated by applying environmental changes, since these induce additional perturbations of gene regulatory networks independently of genetic variants (Fig. 1). Using functional genomics assays in yeast, we confirm that profiling expression in multiple environments is informative in identifying causal intermediates. We anticipate that our observations will facilitate the use of molecular profiling studies to identify causal intermediates that can also serve as intervention points to modulate the effects of genotype on phenotype (Fig. 1).

## Results

**Figure 2:**
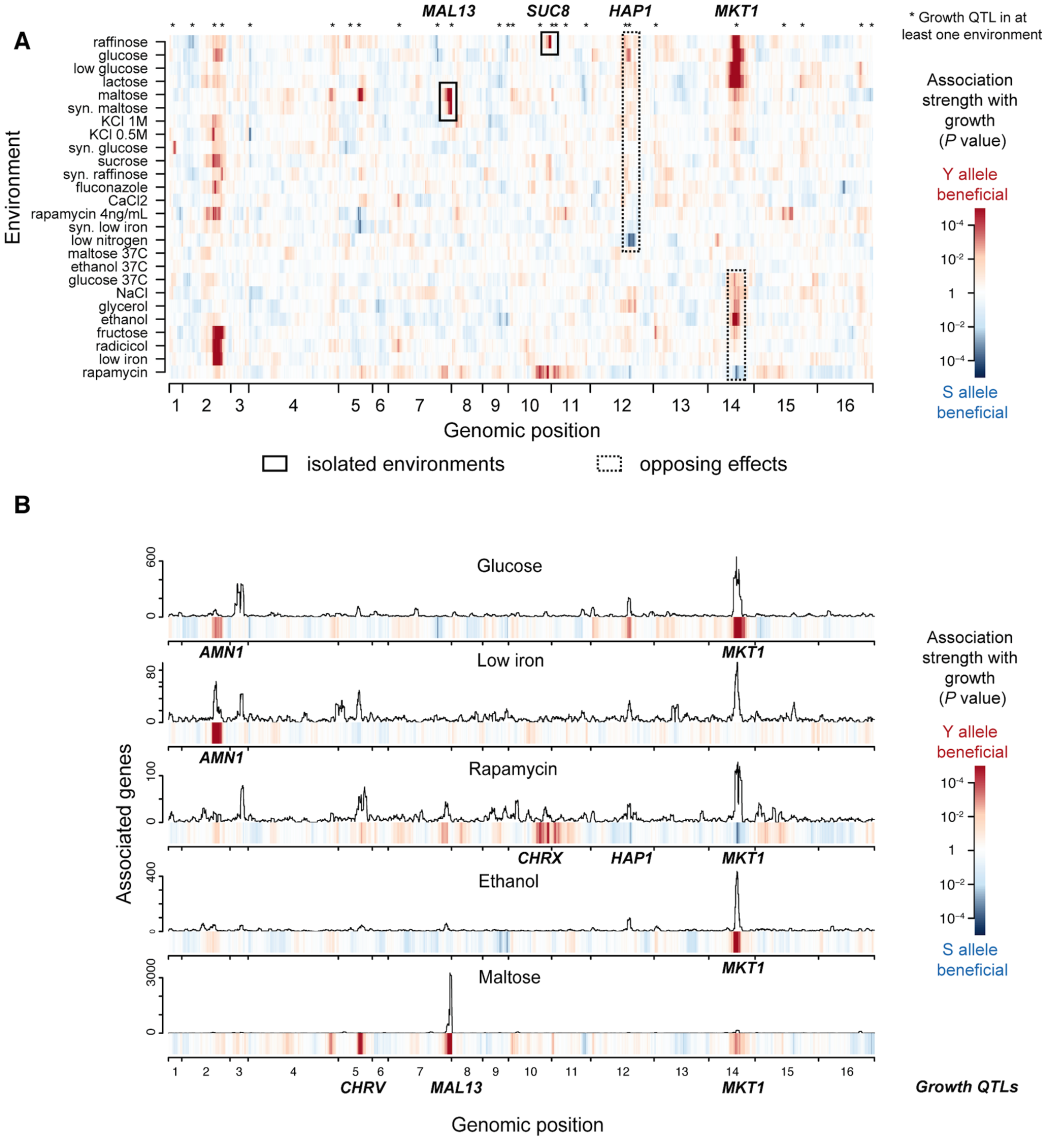
Genetic architecture of growth rate and gene expression in multiple environments. (**a**) Genetic associations with growth (growth QTLs) in 26 environmental conditions. The significance of association (*P*-value, single-marker analysis, Methods) is shown for each of 13,314 markers along the genome (x-axis) with growth rates in 26 environments (y-axis). The direction of the QTL effect is color-coded, where red indicates that the clinical isolate (Y) allele is associated with increased growth rate and blue the lab strain (S) allele; darker colors indicate greater significance. Two examples of markers significantly associated with growth in only a small number of environments (*MAL13* and *SUC8*, black rectangles), and two showing significant effects in opposing directions depending on environment (*HAP1* and *MKT1*, dotted black rectangles) are highlighted. (**b**) Genetic associations with gene expression (eQTLs) in 5 selected environments. Each panel shows the number of genes associated with the underlying regions in a sliding window analysis for each environment (FDR<0.05, 50 kb window). The association strength of growth from **a**) is displayed in the color bars below each panel. Six significant genetic loci were identified that jointly regulate growth in these environments (*AMN1*, *CHRV*, *MAL13*, *CHRX*, *HAP1*, *MKT1*, multi-environment growth genetic model, Methods). These are labeled in bold for every environment in which they were associated with growth in (a) (growth QTL).

To discover causal intermediates for fitness in yeast, we carried out extensive growth and transcriptome profiling of a panel of genetically diverse yeast strains (Table S1). In particular, we monitored the growth of 159 meiotic segregant strains obtained from a cross between a laboratory strain (S96) and a clinical isolate (YJM789) of *Saccharomyces cerevisiae* [12] in 26 diverse environmental conditions (Table S2). We mapped the genetic determinants of growth rate in these environments using genome-wide single marker analysis, which yielded 27 distinct genetic regions (growth quantitative trait loci, QTLs) significantly associated with growth rate in at least one environment (False Discovery Rate, FDR<0.05, Methods, Table S3 and Fig. 2A). Notably, genotype-environment interactions were prevalent, which was reflected in the limited number of growth QTLs that were shared between any pair of environments (15%+/−0.6% standard error of the mean, s.e.m.). Moreover, even shared QTLs varied in the magnitude and, in two instances, in the direction of effects (*i.e.*, which parental allele was associated with faster growth; Fig. 2A): 1) the clinical isolate allele of *MKT1* was detrimental for growth in rapamycin but beneficial in ethanol, glucose and maltose; and 2) the laboratory strain allele of *HAP1* was detrimental in glucose but beneficial in media depleted of nitrogen sources (at FDR<0.1). Together, the prevalence of genotype-environment interactions observed here is in agreement with previous reports ranging from yeast [13] to human [8], demonstrating that genetic effects on phenotype depend heavily on environmental context.

To identify causal intermediates between genotype and growth, we performed transcription profiling in 5 environmental conditions (glucose, low iron, rapamycin, ethanol, maltose; Fig. S1), exceeding the scale of previous studies [6], [8]. These environments were selected from those for which we generated growth profiles, in order to cover all types of genotype-environment interactions encountered in our growth data (including isolated and opposing effects, highlighted in Fig. 2A). We employed a checkered experimental design, whereby random subsets of approximately 35 strains per environment were selected for expression profiling (Fig. S1). This approach offers the advantage that, at a moderate cost, a large proportion of the genetic variation in the entire population is covered. Transcriptome annotation across the 183 tiling microarrays analyzed yielded 8,382 transcribed regions (hereafter called genes, which include coding and non-coding genes; Table S4, Methods). The expression of between 22% (low iron) and 50% (maltose) of these genes was associated with at least one genomic locus (expression quantitative trait locus, eQTL; FDR<0.05; Fig. 2B, Fig. S2). The fraction of shared eQTLs for individual genes between any pair of environments was highly variable (Fig. S3), suggesting that genetic regulation of gene expression can vary in its sensitivity to environmental changes depending on the gene.

Across these 5 environments, the genetic effects on growth rate were captured by 6 major growth QTLs (Fig. 2B: *AMN1*, CHRV, *MAL13*, CHRX, *HAP1*, *MKT1*; determined by multi-environment growth genetic model, Methods). The phenotypic variance explained by this model differed across environments, ranging from 31% in glucose to 52% in maltose (Fig. S4). Each growth QTL was also associated with the expression of a large number of genes in the same environment (*i.e.*, with eQTLs within 50 kb of the growth QTL: Fig. 2B, ranging from 76 genes (0.9%) for *AMN1* in low iron to 2,894 genes (35%) for *MAL13* in maltose). Notably, some of these loci were also associated with gene expression in environments where they were not associated with growth. For example, expression levels of 111 genes were associated with the *MKT1* locus in low iron (within 50 kb, Fig. 2B), although *MKT1* was not associated with growth in that condition. To characterize how environment modulates the effect of growth QTLs on gene expression, we modeled expression levels as the sum of 1) a genetic effect that persists in direction and amplitude across environments (hereafter ‘persistent’), and 2) an environment-dependent effect. This showed that the number and proportion of persistent genes varied greatly across individual growth QTLs and environments (ranging from 0% for *MAL13* in maltose to 86% for *AMN1* in glucose; FDR<0.05; Fig. S5). Thus, transcriptome profiling across 5 environments revealed that growth QTLs affected gene expression in two manners: persistent and, akin to their effects on growth, environment-dependent.

**Figure 3:**
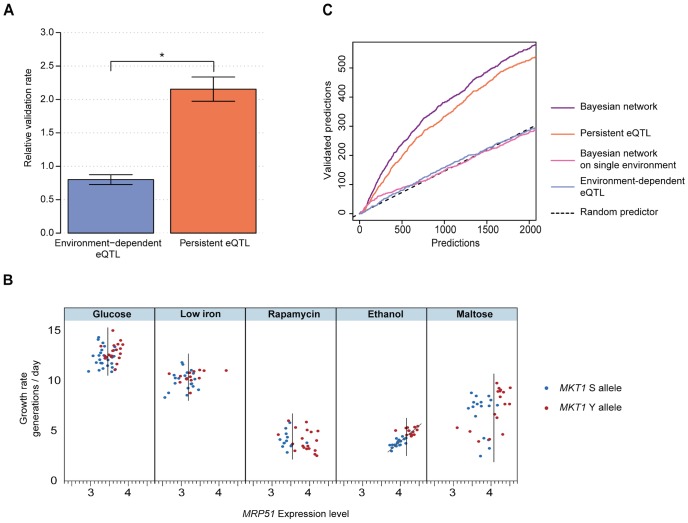
eQTLs that persist across environments are effective predictors of causal intermediates. (**a**) Validation rate (relative to a random selection of genes, Methods) for the top 100 genes whose expression was significantly associated with a growth QTL that is environment-dependent (blue) or persistent (orange), based on genome-wide deletion assays. Error bars indicate plus or minus one standard deviation (jackknife resampling of the growth QTLs, Methods). Star indicates significance of difference (*P*<0.002, two-sided paired Wilcoxon rank sum test). (**b**) *MRP51*: example of a candidate causal intermediate predicted by the Bayesian network to mediate the effect of the *MKT1* genotype on growth in ethanol. In each of the 5 environments (panels), growth rate (y-axis) is plotted vs. *MRP51* expression level (x-axis) and *MKT1* genotype is indicated (clinical isolate allele Y in red, laboratory strain allele S in blue) for all profiled segregants. *MRP51* constitutes a strong candidate causal intermediate because: 1) *MRP51* expression is persistently associated with the *MKT1* genotype, in every environment (vertical bars in each panel mark the midpoint between the expression mean of the two subpopulations); and 2) *MRP51* expression correlates with growth in the ethanol environment (trendline based on linear regression, see also Fig. S12). (**c**) Number of predicted causal intermediate genes validated by deletion (y-axis) vs. number predicted, sorted by prediction confidence (x-axis) for Bayesian network of persistent intermediate genes (purple), persistent eQTL associations (orange), Bayesian network based on single environments (pink), environment-dependent eQTL associations (blue), and random selection (black dashed line). Validations were based on genome-wide deletion phenotypes (Methods).

Both persistent and environment-dependent genes are candidate causal intermediates. To test which candidates indeed play a causal role in growth in the environments of interest, we performed parallel growth assays of a genome-wide deletion collection that covers 4,498 distinct non-essential genes [14] (Methods). As observed in previous studies [15], the phenotypic effects of individual deletions varied across environments, yielding between 938 (glucose) and 1,524 (rapamycin) genes whose deletion was either detrimental or beneficial for growth (FDR<0.05; Fig. S6, Methods). The top-ranking environment-dependent candidates at each growth QTL did not significantly affect growth in that environment when deleted (Fig. 3A; 0.80±0.07 fold enrichment compared to genome-wide background, jackknife resampling, Methods). In contrast, persistent candidates were much more likely to be validated by the deletion assay (Fig. 3A; 2.15±0.18 fold enrichment). This enrichment was robust with respect to choices of cutoffs, and also held for the vast majority of individual growth QTLs and environments (see ‘Benchmarking’ in Methods; Figs. S7, S8). Similar results were obtained when environment-persistent eQTLs were defined from a down-sampled dataset (in which the number of data points matched the average number of measurements in any specific environment), ruling out possible biases due to larger statistical power for detecting persistent vs. environment-dependent associations (Fig. S7 and Text S1). Furthermore, the difference in validation rate did not depend on the effect size of either the eQTLs (Fig. S9) or the growth QTLs (Fig. S10). Finally, the number of persistent eQTLs detected appeared to be independent of the effect size of the corresponding growth QTL (Fig. S11). Hence, the robustness with respect to effect sizes suggests that this finding is likely to translate to growth QTLs with smaller genetic effects, which will become detectable in larger populations [16]. Altogether, these findings indicate that persistent eQTLs are more likely to play causal roles in phenotype than environment-dependent eQTLs.

We then sought to develop a statistical model that leverages this insight to predict causal intermediates. The high validation rate of persistent candidates suggests a regulatory model where QTLs control expression of causal genes, which in turn have environment-dependent effects on phenotype (Fig. 1, node A). Former studies in single environments have shown that joint modeling of genotype, gene expression and physiological phenotype can assist in identifying causal intermediates among eQTLs [17], [18], [19]. These approaches exploit the fact that, in contrast to other genes associated with the QTL, variation in expression of causal intermediates entails variation in the physiological phenotype. We thus extended these principles to multiple environments. For each growth QTL and for each gene, we used Bayesian network modeling to estimate the probability that the expression of that gene causally mediates the environment-dependent genetic effect on growth (Bayesian network, Methods). The Bayesian network models the expression level of candidate causal intermediates as a function of a persistent genetic effect, and the growth rate as a function of the expression of the candidate gene in an environment-dependent fashion. The fit of the data to this model is assessed against a null model, in which the gene is not related to the QTL. This model comparison identifies genes whose expression is both associated with the locus of interest across all environments (*i.e.*, persistent eQTLs) and correlated with growth in the environment of interest (indicating that variation in its expression entails variation in growth rate in this environment). For example, Fig. 3B depicts the expression and growth pattern for a high-ranking gene predicted as a causal intermediate for the effect of the *MKT1* genotype on growth in ethanol: *MRP51* shows persistent eQTL association (segregation of high and low expression levels in each environment, x-axis) and correlation between its expression and growth rate in ethanol (quantitative correlation between expression and growth rate in ethanol; a more detailed visualization of these panels is shown in Fig. S12). Because this particular gene fulfills both of these association patterns with genotype and phenotype, it was among the top ranked candidates. Our deletion assay validated the causative role of *MRP51* expression in conditioning growth in ethanol.

To evaluate the performance of the Bayesian network genome-wide, we compared its predictions to our deletion validations. The deletion screen supported 50% of the top 422 predictions across all growth QTLs. Furthermore, the Bayesian network (Fig. 3C, purple) that combines genotype, gene expression, and growth rate was consistently more accurate than predictions based on persistent expression associations alone that do not consider growth (Fig. 3C, orange). To compare our approach with previous attempts to infer causal relationships in genetic networks that analyzed only one environment [17], [18], [20], we constructed an analogous Bayesian network that restricts the analysis to the specific environment with a growth QTL. These single-environment approaches, either by basic eQTL association or by Bayesian network modeling, did not yield meaningful accuracy (Fig. 3C, blue and pink), underscoring the value of integrated analysis across multiple environments for predicting causal intermediates.

Functional annotations of the predicted causal intermediate genes were enriched in molecular pathways related to the environment of interest and the underlying genetic variant for most growth QTLs (Table S4 and Methods). For the *MAL13* growth QTL, which encodes a regulator of the maltose pathway [21] and whose genetic influence on growth in maltose we validated by reciprocal hemizygosity analysis (Methods and Fig. S13), our model predicted two genes (*MAL31*, and a non-coding RNA *SUT145*). In support of this prediction, overexpression of *MAL31* has been shown to rescue growth in maltose for the laboratory strain [22]. The functional specificity of this prediction, identifying just two genes, is in stark contrast to the 2,894 genes whose expression level was associated with the *MAL13* genotype in maltose (FDR<0.05, within 50 kb; Fig. 2B). Since the majority of these 2,894 genes were not functionally validated by our deletion assay (12.0% validation rate vs. 11.2% genome-wide), most of them may well be consequences of the pronounced variation in growth rates (*e.g.*, Figure 1, nodes E, F). Altogether, these data show that regulatory effects on whole pathways can occur persistently across multiple environments, even though their functional impact on growth is apparent only in specific environments. Therefore, the integration of multiple environmental conditions improves the prediction of causal intermediates that transmit genetic effects to phenotype.

**Figure 4:**
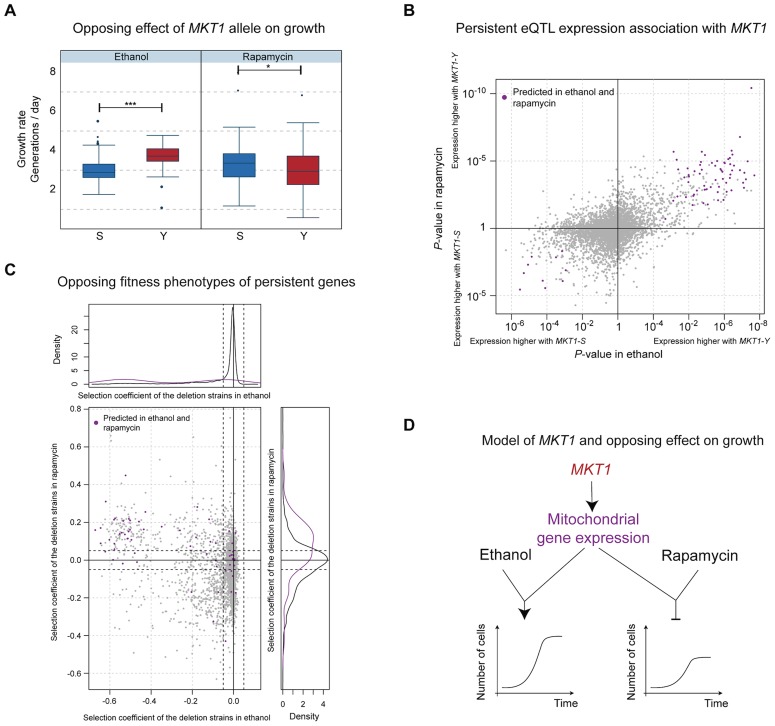
Molecular basis of the *MKT1* genotype's opposing effects on growth. (**a**) Distribution of growth rates according to *MKT1* genotype (laboratory strain allele (S) in blue, clinical isolate allele (Y) in red) in two environments: ethanol (left panel) and rapamycin (right panel). The association is significant for both environments (*P*<6×10^−10^ and *P*<0.05 respectively, two-sided Wilcoxon rank sum test) but the alleles have opposing effects on growth (S detrimental in ethanol, Y detrimental in rapamycin). (**b**) Association of *MKT1* genotype with gene expression (*P*-value, right and top display higher expression values associated with the clinical strain allele, left and bottom display higher expression levels associated with the laboratory strain allele) in ethanol (x-axis) and rapamycin (y-axis) for all genes. Unlike for growth, the overall effects of *MKT1* genotype on gene expression levels are in the same direction (positive correlation; Wilcoxon rank-sum test P<2.2×10^−16^). High-ranking candidate causal intermediate genes according to the Bayesian network and common to both environments (89 common genes from the top 100 in each environment) are highlighted in purple. (**c**) Fitness defects induced by gene deletion (selection coefficient from deletion collection assay, Methods) in ethanol (x-axis) versus rapamycin (y-axis), color-coded as in b). Left and upper panels show the distribution of the selection coefficient for the deletion of the candidate genes (purple) and all other genes (grey). Candidate genes (purple) are typically beneficial for growth in ethanol and detrimental in rapamycin. (**d**) Model of the genotype-environment interaction that explains the *MKT1* genotype's opposing effects on growth. The *MKT1* clinical isolate allele upregulates expression of several mitochondrial genes (Table S4) regardless of environment; this regulation leads to improved growth rates in ethanol, but repressed growth in the presence of rapamycin.

Predictions from our model provided a mechanistic explanation for the opposing effects associated with the *MKT1* locus. The clinical isolate allele resulted in increased fitness in several environments, most pronounced in ethanol, but decreased fitness in rapamycin (Fig. 4A). In contrast, *MKT1* genetic effects on gene expression levels in these environments tended to be consistent in direction and amplitude, indicating that the genetic variant affects the same network of genes irrespective of the environment (Fig. 4B; positive correlation, Wilcoxon rank-sum test *P*<2.2×10^−16^). Likewise, the majority of the candidate causal intermediates predicted in ethanol and rapamycin were shared (89 of the top 100 in each environment). These genes have been implicated in mitochondrial function (Table S5) and higher expression levels were typically associated with the clinical isolate allele in both environments (Fig. 4B, top-right quadrant). Deletion of these predicted causal genes typically resulted in impaired growth in ethanol and improved growth in rapamycin (Fig. 4C, top-left quadrant). These findings are consistent with (i) regulation of mitochondria-localized genes by *MKT1* [23], (ii) the well-characterized role of mitochondria in growth on non-fermentable media such as ethanol, and (iii) a previous report of nine mitochondrial genes as being detrimental to survival in rapamycin [24]. Altogether, these results explain the molecular basis of the opposing effects of *MKT1* on growth rate in different environments (Fig. 4D). They also confirm that genetic effects on causative molecular pathways can occur in multiple environments, yet the functional impact of these pathways on phenotype may still be environment-dependent.

## Discussion

Our results demonstrate that the integration of genetic and environmental variation into molecular profiling efforts improves the identification of causal intermediates. Why is this improvement so pronounced? Environmental cues trigger molecular processes that perturb the chain of molecular events linking genotype to phenotype. Our findings indicate that the immediate molecular consequences of DNA variation, lying furthest upstream in this cascade, are less likely to depend on environment (Fig. 1 nodes A, C). In contrast, the events furthest downstream of genotype, including side effects and consequences of phenotypic changes, are most often environment-dependent (Fig. 1a, nodes B, E, F, G). Our data show that causal intermediates can be effectively identified among the most upstream molecular players, such as genes whose expression is persistently associated with genetic variants across multiple environments.

The systematic deletion assay was instrumental for validating strategies to predict causal intermediates. The limitations of this approach are that it does not detect combinatorial effects and may miss complex genetic dependencies since the deletions are made in only one parental background. Additional functional assays, for example from double-knockout experiments, could be used to refine the validation information by tackling combinatorial effects. Nevertheless, we were able to identify a large number of genes within causal intermediate pathways for each growth QTL. Our dataset thus constitutes a useful reference for developments of novel causal inference methods.

Analogous to growth in yeast, genetic predisposition to disease is mediated by gene expression and depends on cellular context, including environment, tissue, and cell type. We observed that both persistent and context-dependent eQTLs are common and frequently occur at genetic loci that affect physiological phenotypes, consistent with previous reports from yeast [6] to human [8]. Moreover, most of our validated causal intermediates were not located in the vicinity of the growth QTL (203 of the 211 validated candidates at 50% precision cutoff were on another chromosome), verifying that our approach captures more than the direct *cis*-regulatory consequences of genetic variants. The identification of *trans* acting intervention points is important, as it yields larger sets of possible intervention points and allows for addressing QTLs located within gene deserts, like those frequently reported in genome-wide association studies. With larger sample sizes, *trans* associations are also increasingly being detected in human [4], although with weaker effect sizes. We have confirmed that the basic principles we discovered in yeast are robust with respect to the effect size of the association with phenotype and the genetic effect on gene expression. Hence, although the experimental design of a cross is specific to model organisms, our reported results and conclusions should also hold in higher eukaryotes.

In particular, our findings have implications for the experimental design of omics profiling of large clinical cohorts. Previous studies have suggested that disease-afflicted tissues are most informative in molecular profiling efforts, since they should more comprehensively capture the molecular consequences of genetic defects [11]. Our results suggest otherwise: it may be more difficult to distinguish causal regulatory changes from their consequences in affected tissues, perhaps because consequences of phenotypes will be more prevalent in these tissues. To disentangle causes and consequences, therefore, our findings attest to the utility of molecular profiling in diverse contexts, even if the overall number of profiling experiments is not increased. This includes longitudinal studies of individuals that carry a genetic defect before complex symptoms arise, and the profiling of matched control samples of the same tissue type that are not affected by the disease. Our results obtained with a checkered random design suggest that incomplete data, as commonly encountered in clinical settings, can be effectively analyzed to yield genuinely causal insights.

Such experimental designs in conjunction with causal inference algorithms as developed here can help to reveal key associations that indicate pathways with a causal role in the progression of genetic disease. Exploring strategies to leverage these mechanistic insights to develop treatments will be an important direction for systems medicine research.

## Materials and Methods

### Data availability

The data reported in this paper have been deposited in the ArrayExpress repository (http://www.ebi.ac.uk/arrayexpress/) under accession number E-MTAB-1398.

### Strains, media, and primers

The segregants consist of 159 of the 184 segregants previously derived from a cross of *S. cerevisiae* strains S96 (MATa ho:: lys5 gal2) and YJM789 (MATα ho::hisG lys2 gal2) (see [12] and Table S1). Further strains were generated to confirm the *MAL13* growth QTL (SI Methods). The complete list of growth media is given in Table S2. Primers are listed in Table S6.

### Growth profiling

Strains were grown and their optical densities were tracked in a TECAN GENios multiwell plate reader. For the five environments of focus, measurements were repeated in triplicates and alternative layouts were compared. Growth rates were estimates using the R/Bioconductor cellGrowth package (SI Methods).

### Transcription profiling and annotation

The segregants were grown at 30°C to mid-exponential phase. Tiling-array based transcription profiling was done as previously described [25] and applied to a random subset of strains in all environments, resulting in 184 arrays overall. Normalization included variance stabilization and an additional quantile normalization step (Text S1). Gene expression levels were estimated from a robust average across probes, accounting for overlapping genes. Transcriptome annotation was carried out jointly across all environments (Text S1).

### Deletion collection profiling

Aliquots of the deletion collection were obtained from Robert St. Onge (Stanford Genome Technology Center, Palo Alto, CA). After overnight growth at 30°C, triplicates comparing relative abundances for barcoded deletion strains at generation 5 and generation 0 were profiled(Text S1). For each strain, the selection coefficient (or relative growth rate) was estimated using a linear model of log hybridization intensity and its significance assessed with a moderated t-test (Text S1).

### Correction for multiple testing

False Discovery Rates were estimated according to the Storey and Tibshirani procedure [26].

### eQTL mapping

Standard single-marker analysis was used, testing individual genetic variants for association with expression or growth phenotypes in a specific environment. To account for non-i.i.d. sample structure caused by the checkered experimental design, all association analyses were done using a linear mixed model, similar to EMMA [27] with a random effect that corrects for genotype structure (Text S1). Environment-dependent versus persistent eQTLs were classified by joint analysis across all environments, considering a shared main effect and interaction term in a particular environment. The joint growth genetic model was derived by means of stepwise regression (Text S1).

### Bayesian network

To predict causal intermediate genes, we first fit a joint genetic growth model (Text S1). Next, we considered each gene-environment interaction term in this growth genetic model and tested each gene for mediating its effect [17], [18], [19]. This test was carried out by comparing two Bayesian networks that assume a mediating role (causal intermediate gene) versus no mediation. Let **g** be the vector of the growth rates in all samples, **t** the vector of gene expression of the gene of interest, **s** the genotype indicator matrix and **E** the environment indicator matrix. The two models compared by our approach can be specified as follows. In Model 1 (causal intermediate gene), growth rate and genotype at the interaction marker are assumed to be independent conditioned on gene expression. Furthermore, the gene is under environmentally persistent regulation of the corresponding marker. For a particular interaction term (*n_i_*, *e_i_*,), the joint distribution encoding these statistical dependencies is:
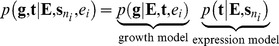
n Model 0 (or null Model), gene expression is assumed to be independent of the growth QTL genotype and the model for the growth rate is identical to the multi-environment growth genetic model:
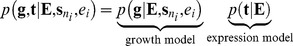
Model 0 and Model 1 were parameterized as linear Gaussian models (Text S1). Model comparison using Bayesian Information Criterion was carried out to estimate the posterior probability of Model 1 (causal intermediate gene) for any particular gene. All terms of the growth genetic model except the one considered were included as covariates (Text S1).

### Deletion benchmarking

eQTL mapping and predictions from the Bayesian network were assessed in terms of their ability to predict genes with a functional effect on growth in the relevant environments as identified from the deletion collection profiling data. All assessments were done on the subset of 4,498 non-essential genes. The direction of the effect (beneficial or detrimental for growth) was deduced from the correlation between gene expression and growth and included in the evaluation (Text S1).

Full methods, including a more detailed description of the statistical analyses, are provided in Supplementary Information (Text S1).

## Supporting Information

Figure S1 Checkered experimental design used for expression profiling. In each environment (columns), approximately equal sized fractions of 32 (Rapamycin) to 35 (Ethanol) and 48 (Glucose) segregants (rows) were randomly selected (red rectangles) for expression profiling.
(PDF)

Figure S2 Distribution of eQTL per environmental condition. Left panel: Number of significant eQTLs (single marker analysis FDR<0.05) per condition and distance of associated marker to expressed gene (distal if more than 25 kb away, dark grey and local otherwise, light grey). Right panel: number of distinct genes with at least one significant eQTL per condition.
(PDF)

Figure S3 Distribution of the fraction of shared eQTLs between any pair of the five environments. For reference, the bar indicates the fraction of sharing for the growth phenotype in these five environments (18%+/−2%), which is similar to the sharing in the full growth panel across 26 environments (15%+/−0.6%).
(PDF)

Figure S4 Fraction of variance explained by genotype. For each environment (YPD,…, YPMalt, see Table S2) the fraction of phenotypic variance explained by the terms fit in the joint growth genetic model (black bar). For reference, a richer model that includes a polygenic background of all variants except those in the growth genetic model is included (grey).
(PDF)

Figure S5 Distribution of eQTL associations at growth QTLs. For each environment (YPD, …, YPMalt, see Table S2) and growth QTLs (*AMN1*, …, *MKT1*), the total number of significant eQTLs (single marker analysis, FDR<0.05) are broken down into relative fractions of different categories: those with only a significant environment-dependent association (blue), only a significant environment-persistent association (orange) or both (green). Absolute numbers of eQTLs in each category are shown above each bar. In order to maintain comparable statistical power for both categories, persistent associations have been computed from a sub-sampled dataset, such that the number of data points matches the average number of measurements in any specific environment.
(PDF)

Figure S6 For each environment, shown is the absolute number of deletion strains with a significant (FDR<0.05) effect on growth with either a positive selection coefficient (*s*>0.05, orange, improved growth) or negative selection coefficient (*s*<−0.05, green, impaired growth).
(PDF)

Figure S7 Number of validated predictions of causal intermediate genes (y-axis) versus the number of predicted causal intermediate genes sorted by signed prediction (See Text S1) (x-axis) for alternative methods. Considered are environment-persistent eQTL associations (orange), environment-persistent eQTL associations in a randomly selected subsample of the data (orange and dashed), environment-dependent eQTL associations (blue), and random guessing (black dashed line). Sub-sampling (orange dashed) of 35 randomly selected data points, matching the number of samples in individual environments, was done to control for effective sample size differences between tests for persistent and dependent associations.
(PDF)

Figure S8 Rate of functional validation considering the yeast deletion collection for each growth QTL, considering either environment-persistent eQTLs or environment-dependent eQTLs in association with the identical loci. For each environment (YPD,…,YPMalt, see Table S2) and corresponding growth QTL (*AMN1*,…, *MKT1*), shown is the validation rate (relative to a random selection of genes in same environment) of the 100 top ranking associations that are either consistent with genes impairing growth when deleted (upper panel) or predictive to improve growth (lower panel). The total number of genes in each category is shown above each bar (in total about 50 at each growth QTL, since about half of all annotated genes have a matching deletion strain).
(PDF)

Figure S9 Fraction of validated predictions (y-axis) of candidate mediating genes for environment-dependent eQTLs (blue, FDR<0.05) and environment-persistent eQTLs (orange, FDR<0.05, identified at equivalent sample size, see Text S1) stratified by the eQTL effect (log_2_ fold change of expression, x-axis). Error bars show two times standard error of the mean, the number of genes in each category is displayed beneath the bar (*n* = …). Stars indicate significant differences between the two eQTL types (two-sided Fisher test P<0.01) regardless of effect size.
(PDF)

Figure S10 Fraction of validated predictions (y-axis) of candidate mediating genes for environment-dependent eQTLs (blue, FDR<0.05) and environment-persistent eQTLs (orange, FDR<0.05, identified at equivalent sample size, see Text S1) stratified by the growth QTL effect (generations per day, x-axis). The bins have been chosen to contain similar number of QTLs (3, 3 and 4 QTLs respectively). Error bars show two times standard error of the mean, the number of genes in each category is displayed beneath the bar (*n* = …). Stars indicate significant differences between the two eQTL types (two-sided Fisher test P<0.01) regardless of effect size.
(PDF)

Figure S11 Number of persistent eQTL associations (y-axis, FDR<0.05, identified at equivalent sample size, see Text S1) versus the growth QTL effect (generations per day, x-axis).
(PDF)

Figure S12 *MRP51*: example of a candidate gene predicted by the Bayesian network to mediate the effect of *MKT1* genotype on growth in ethanol. This corresponds to Figure 3B with a separate y-axis scale for each panel to better show the behavior in the Ethanol environment. In each of the 5 environments (panels), growth rate (y-axis) is plotted vs. expression levels (x-axis) and *MKT1* genotype is indicated (clinical isolate allele red, laboratory strain allele blue) for all profiled segregants. *MKT1* genotype displays persistent associations with *MRP51* expression: the latter segregates with the *MKT1* genotype in every environment (vertical bars in each panel mark the midpoint between the expression mean of the two subpopulations). Expression correlates with growth in the Ethanol environment (trend line based on linear regression); *MRP51* thereby fulfills all the criteria for a causal intermediate transcript.
(PDF)

Figure S13 Distribution of growth rate (in generations per day, y-axis) for the hybrid cross between the lab strain and the clinical isolate (S96×YJM789, *n* = 4), for the hybrid cross where the reference strain allele of *MAL13* is deleted (S96dMAL13×YJM789, *n* = 12) and the hybrid cross where the clinical isolate strain allele of *MAL13* is deleted (S96×YJM789dMAL13, *n* = 6). The latter two differ significantly in growth rate (P<0.001, one-sided Wilcoxon rank sum test).
(PDF)

Table S1 Strains.
(TXT)

Table S2 Growth media.
(TXT)

Table S3 Growth QTLs.
(TXT)

Table S4 Genes.
(TXT)

Table S5 Gene set enrichment for candidate mediating genes.
(TXT)

Table S6 Primers.
(TXT)

Text S1 Supplementary information.
(PDF)
