# Supplementary figures and images for: Genotype-Environment Interactions Reveal Causal Pathways That Mediate Genetic Effects on Phenotype
Source: PLoS Genet. 2013 Sep 19;9(9):e1003803. doi: 10.1371/journal.pgen.1003803 (PMC3778020; doi:10.1371/journal.pgen.1003803)

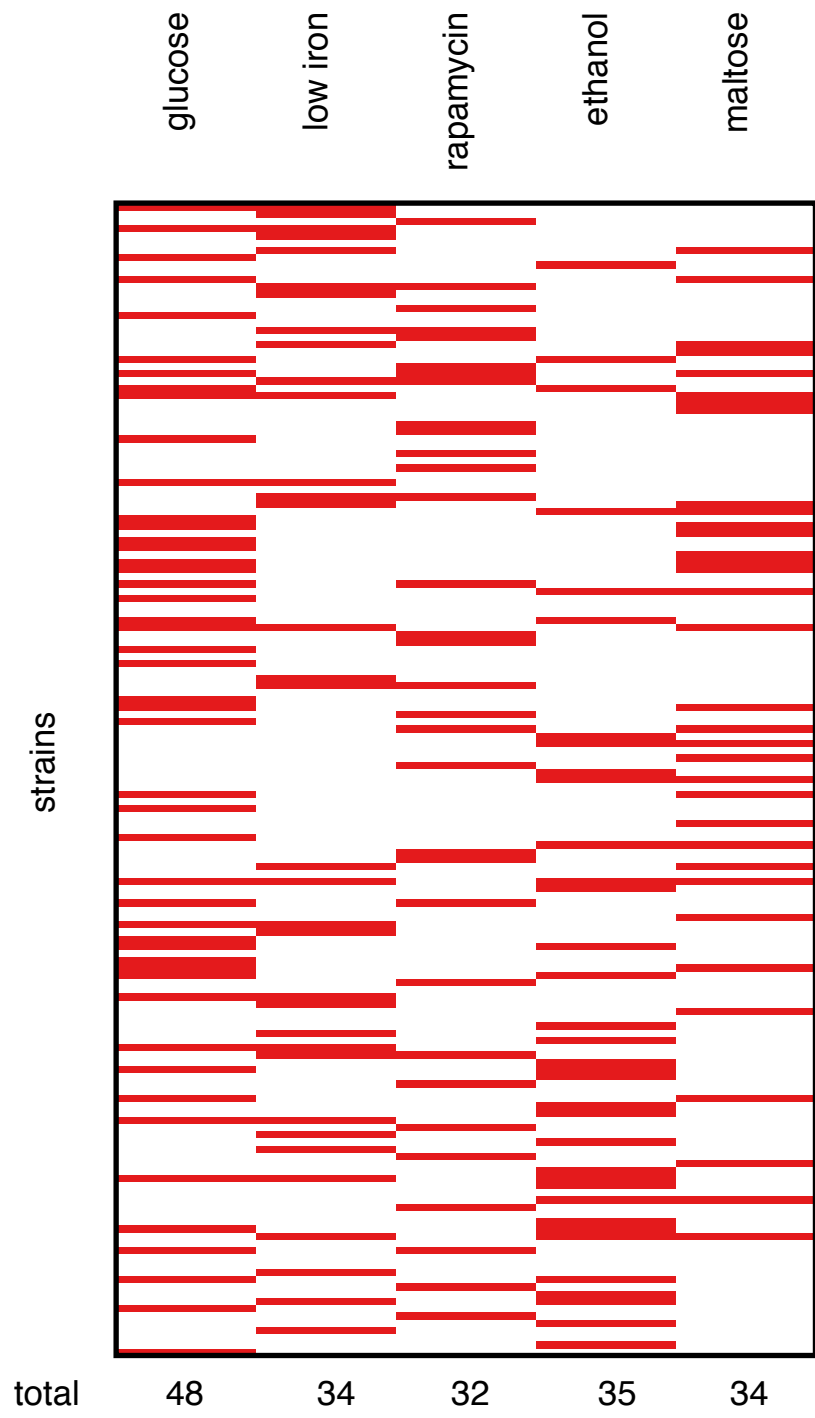

Supplement: Figure S1 — Checkered experimental design used for expression profiling. In each environment (columns), approximately equal sized fractions of 32 (Rapamycin) to 35 (Ethanol) and 48 (Glucose) segregants (rows) were randomly selected (red rectangles) for expression profiling. (PDF) [file pgen.1003803.s001.pdf]

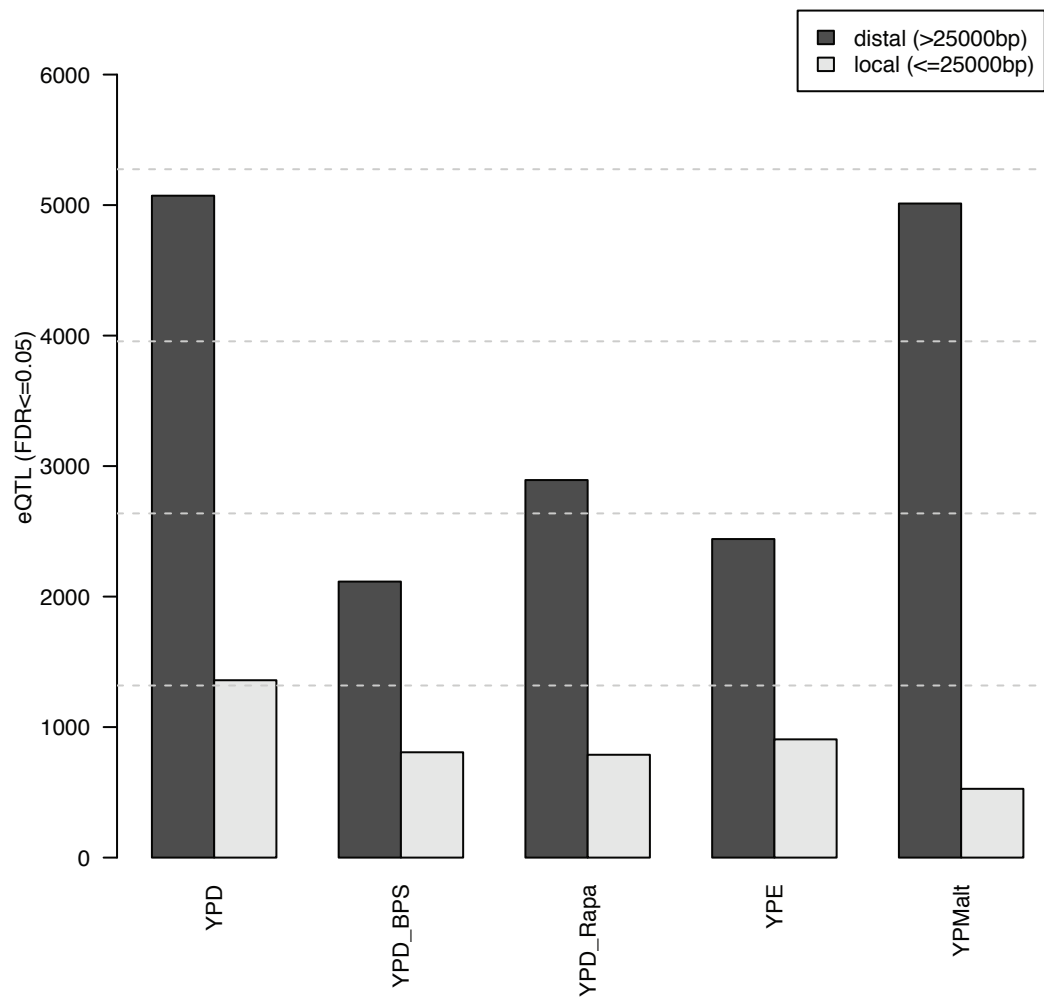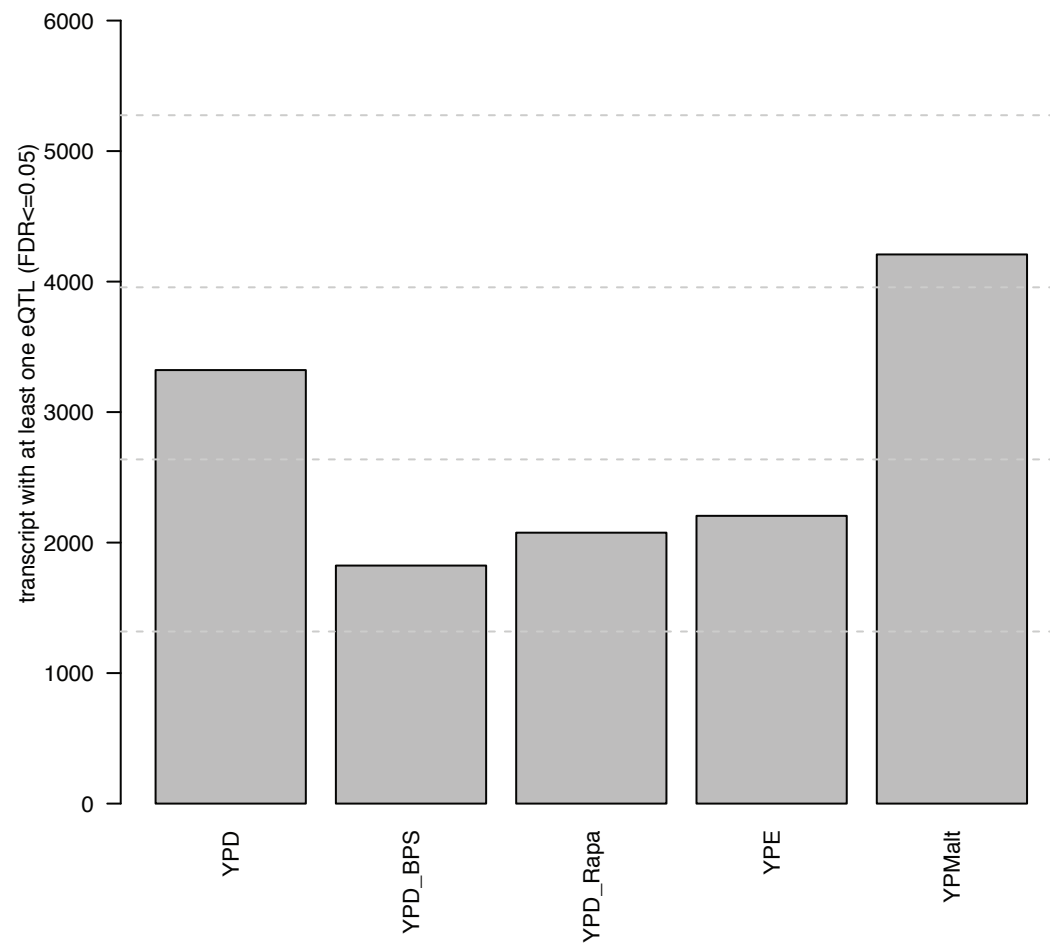

Supplement: Figure S2 — Distribution of eQTL per environmental condition. Left panel: Number of significant eQTLs (single marker analysis FDR<0.05) per condition and distance of associated marker to expressed gene (distal if more than 25 kb away, dark grey and local otherwise, light grey). Right panel: number of distinct genes with at least one significant eQTL per condition. (PDF) [file pgen.1003803.s002.pdf]

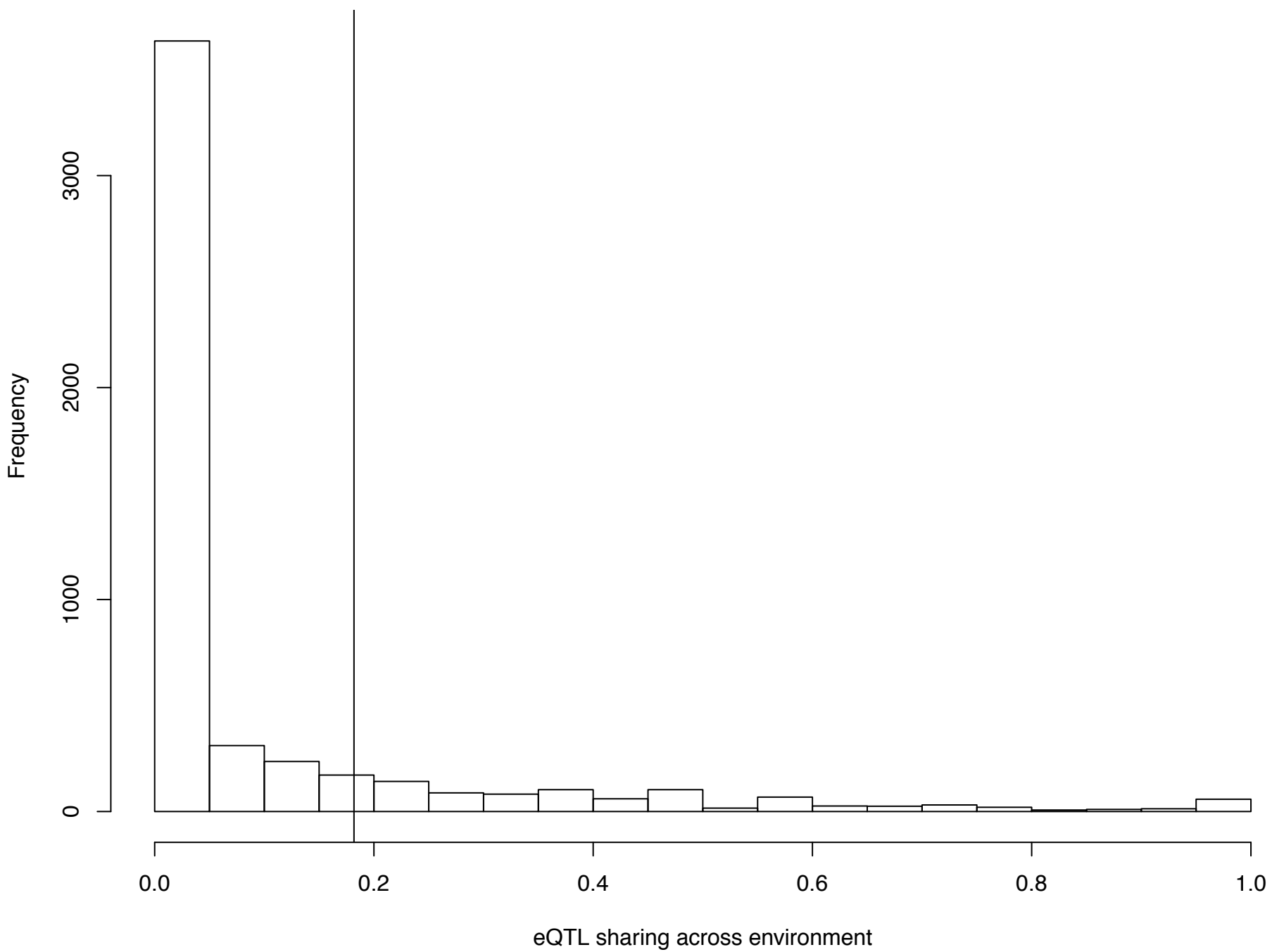

Supplement: Figure S3 — Distribution of the fraction of shared eQTLs between any pair of the five environments. For reference, the bar indicates the fraction of sharing for the growth phenotype in these five environments (18%+/−2%), which is similar to the sharing in the full growth panel across 26 environments (15%+/−0.6%). (PDF) [file pgen.1003803.s003.pdf]

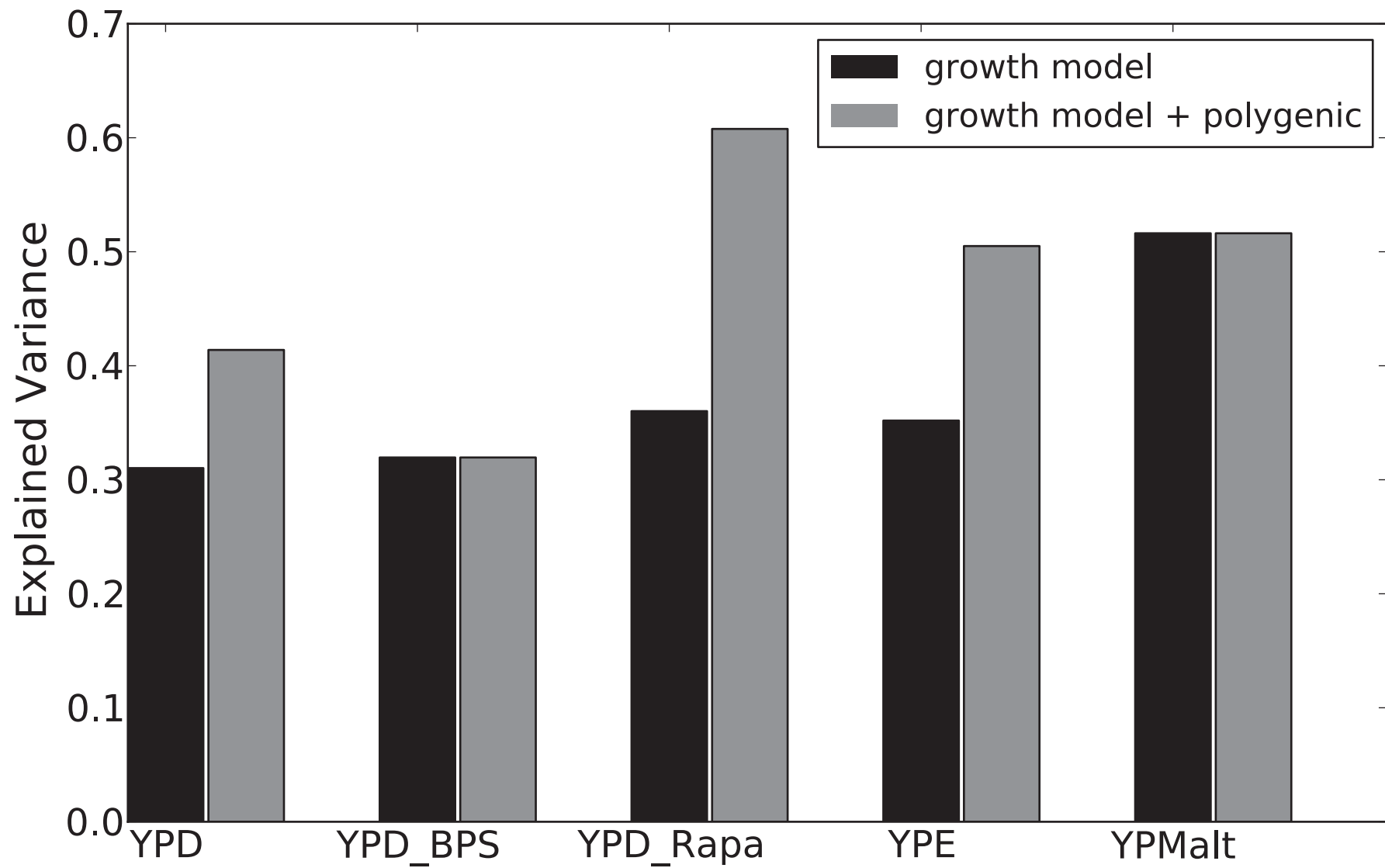

Supplement: Figure S4 — Fraction of variance explained by genotype. For each environment (YPD,…, YPMalt, see Table S2) the fraction of phenotypic variance explained by the terms fit in the joint growth genetic model (black bar). For reference, a richer model that includes a polygenic background of all variants except those in the growth genetic model is included (grey). (PDF) [file pgen.1003803.s004.pdf]

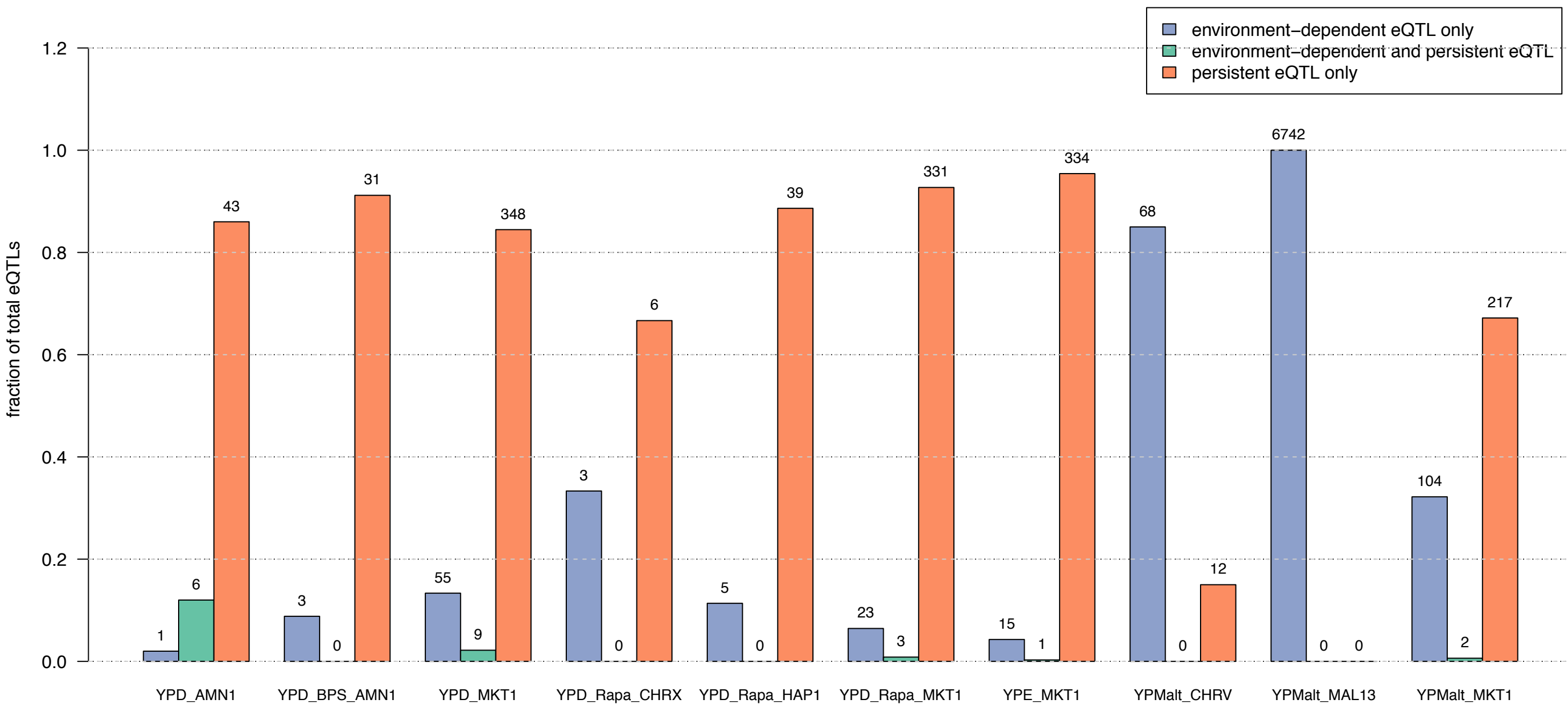

Supplement: Figure S5 — Distribution of eQTL associations at growth QTLs. For each environment (YPD, …, YPMalt, see Table S2) and growth QTLs (AMN1, …, MKT1), the total number of significant eQTLs (single marker analysis, FDR<0.05) are broken down into relative fractions of different categories: those with only a significant environment-dependent association (blue), only a significant environment-persistent association (orange) or both (green). Absolute numbers of eQTLs in each category are shown above each bar. In order to maintain comparable statistical power for both categories, persistent associations have been computed from a sub-sampled dataset, such that the number of data points matches the average number of measurements in any specific environment. (PDF) [file pgen.1003803.s005.pdf]

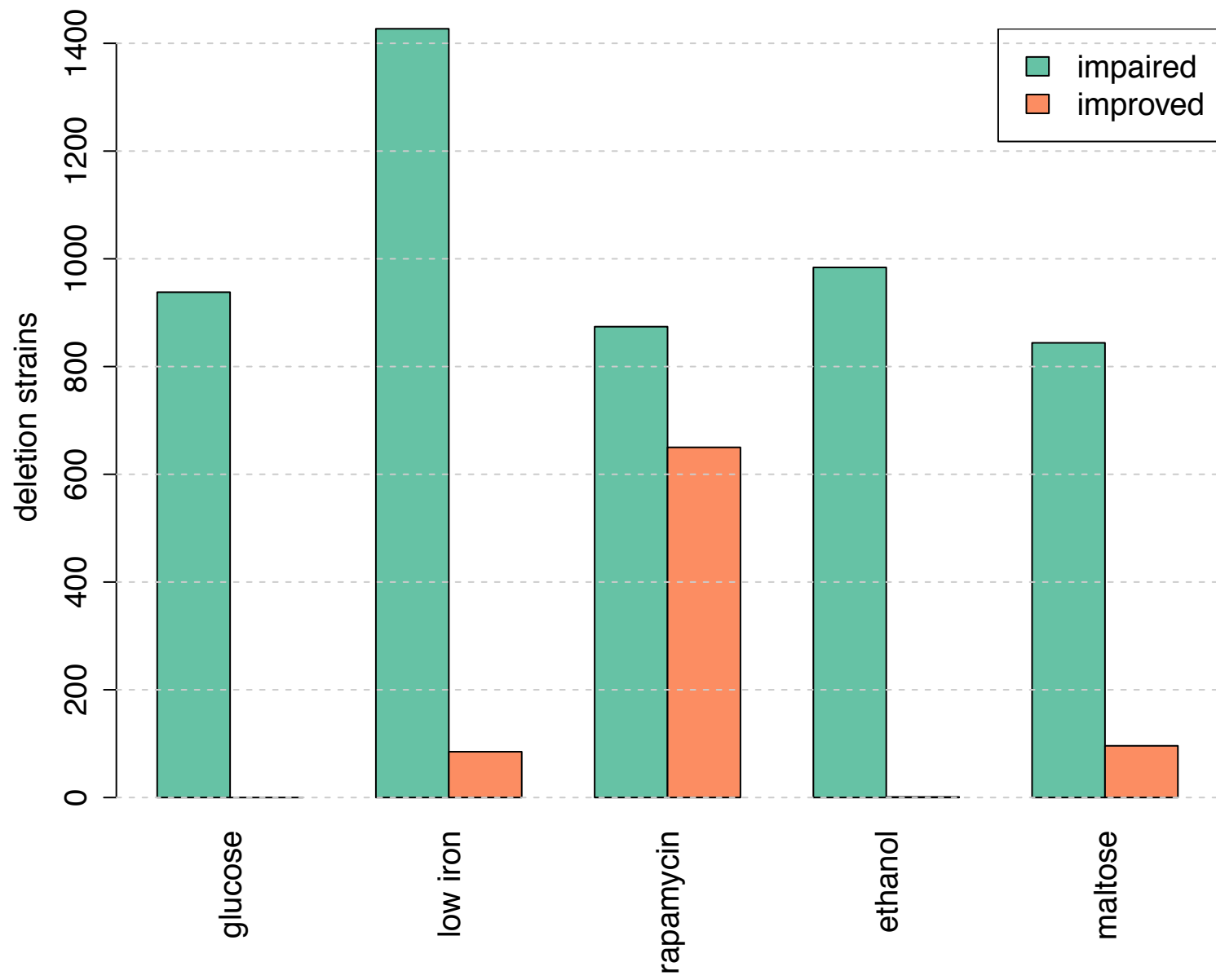

Supplement: Figure S6 — For each environment, shown is the absolute number of deletion strains with a significant (FDR<0.05) effect on growth with either a positive selection coefficient (s>0.05, orange, improved growth) or negative selection coefficient (s<−0.05, green, impaired growth). (PDF) [file pgen.1003803.s006.pdf]

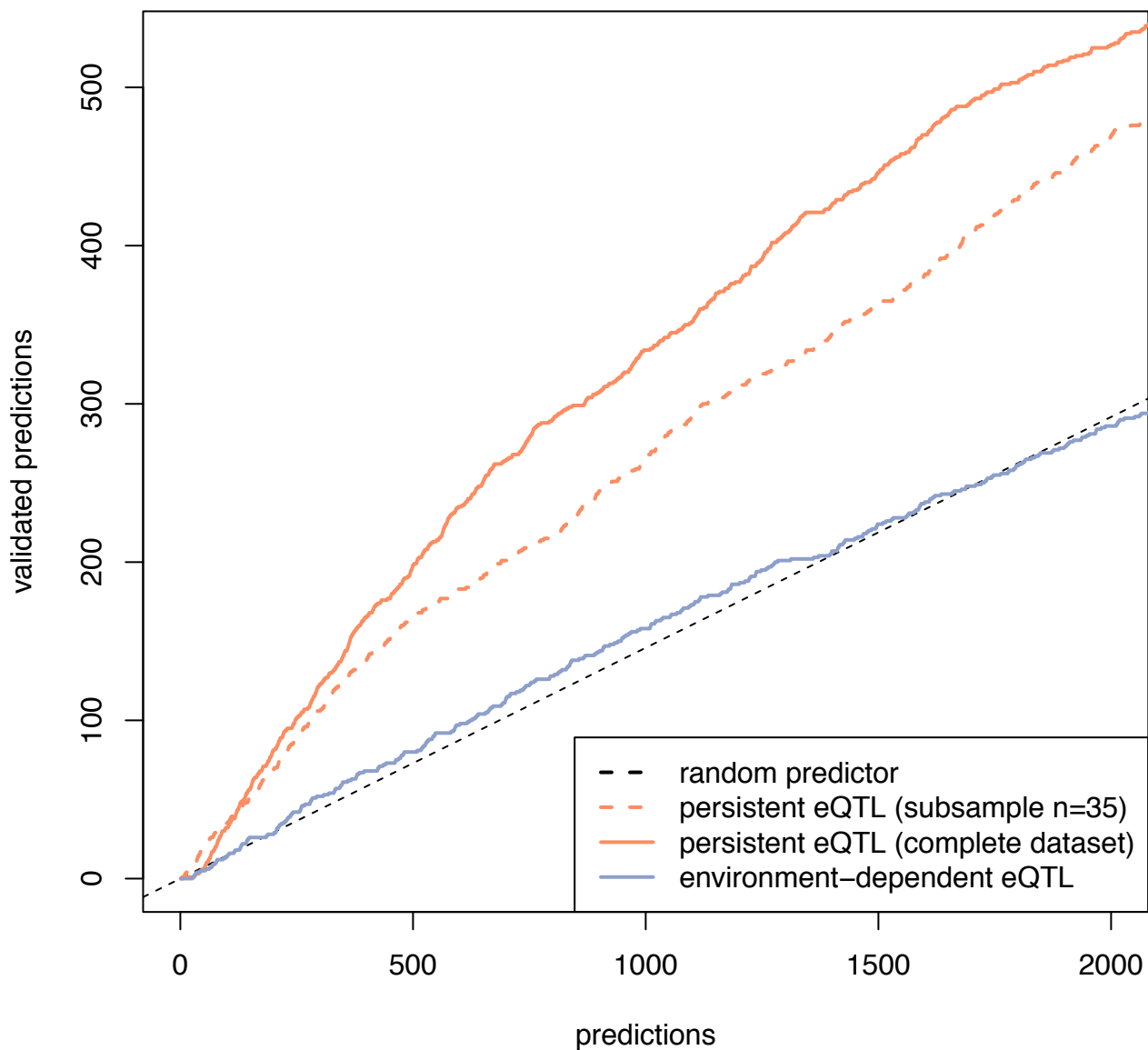

Supplement: Figure S7 — Number of validated predictions of causal intermediate genes (y-axis) versus the number of predicted causal intermediate genes sorted by signed prediction (See Text S1) (x-axis) for alternative methods. Considered are environment-persistent eQTL associations (orange), environment-persistent eQTL associations in a randomly selected subsample of the data (orange and dashed), environment-dependent eQTL associations (blue), and random guessing (black dashed line). Sub-sampling (orange dashed) of 35 randomly selected data points, matching the number of samples in individual environments, was done to control for effective sample size differences between tests for persistent and dependent associations. (PDF) [file pgen.1003803.s007.pdf]

### deletion predicted to impair growth in media

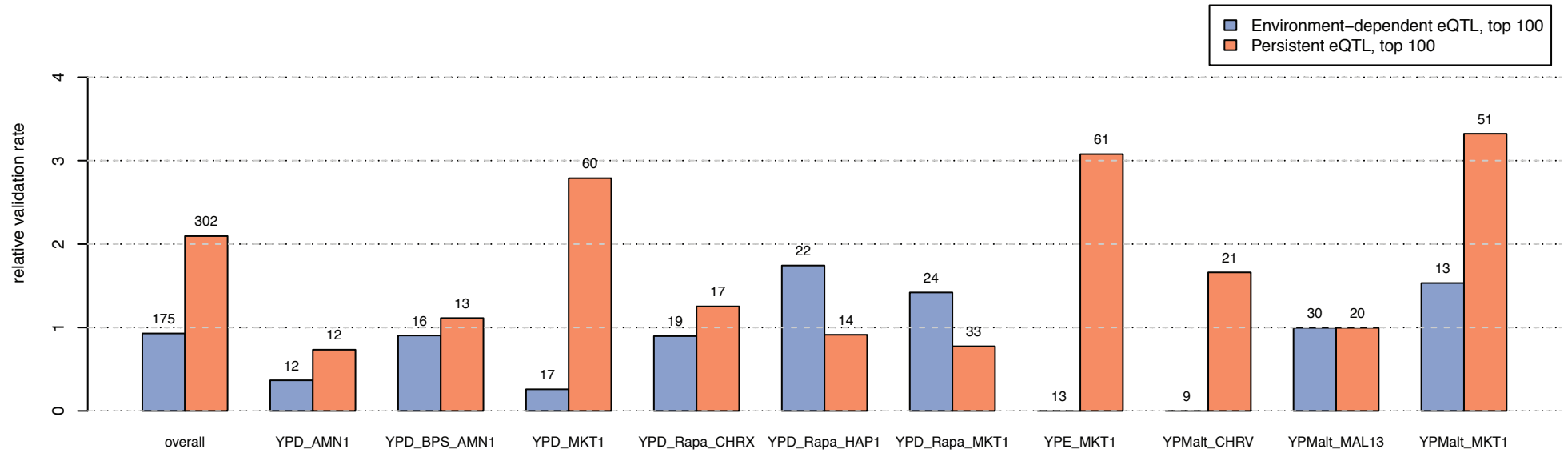

### deletion predicted to improve growth in media

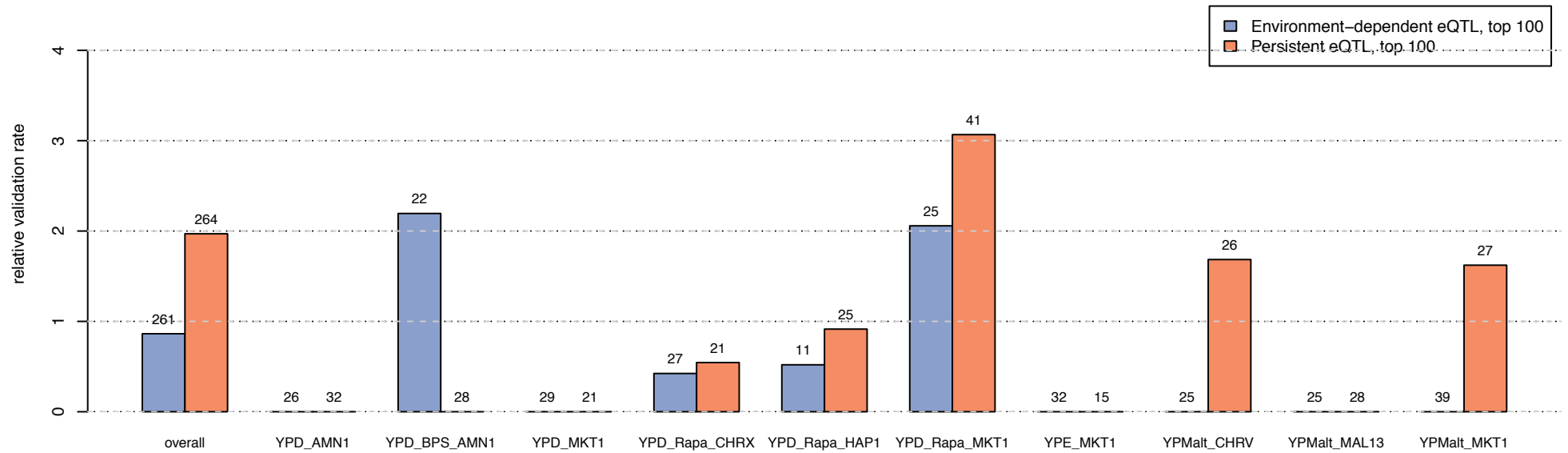

Supplement: Figure S8 — Rate of functional validation considering the yeast deletion collection for each growth QTL, considering either environment-persistent eQTLs or environment-dependent eQTLs in association with the identical loci. For each environment (YPD,…,YPMalt, see Table S2) and corresponding growth QTL (AMN1,…, MKT1), shown is the validation rate (relative to a random selection of genes in same environment) of the 100 top ranking associations that are either consistent with genes impairing growth when deleted (upper panel) or predictive to improve growth (lower panel). The total number of genes in each category is shown above each bar (in total about 50 at each growth QTL, since about half of all annotated genes have a matching deletion strain). (PDF) [file pgen.1003803.s008.pdf]

validation rate

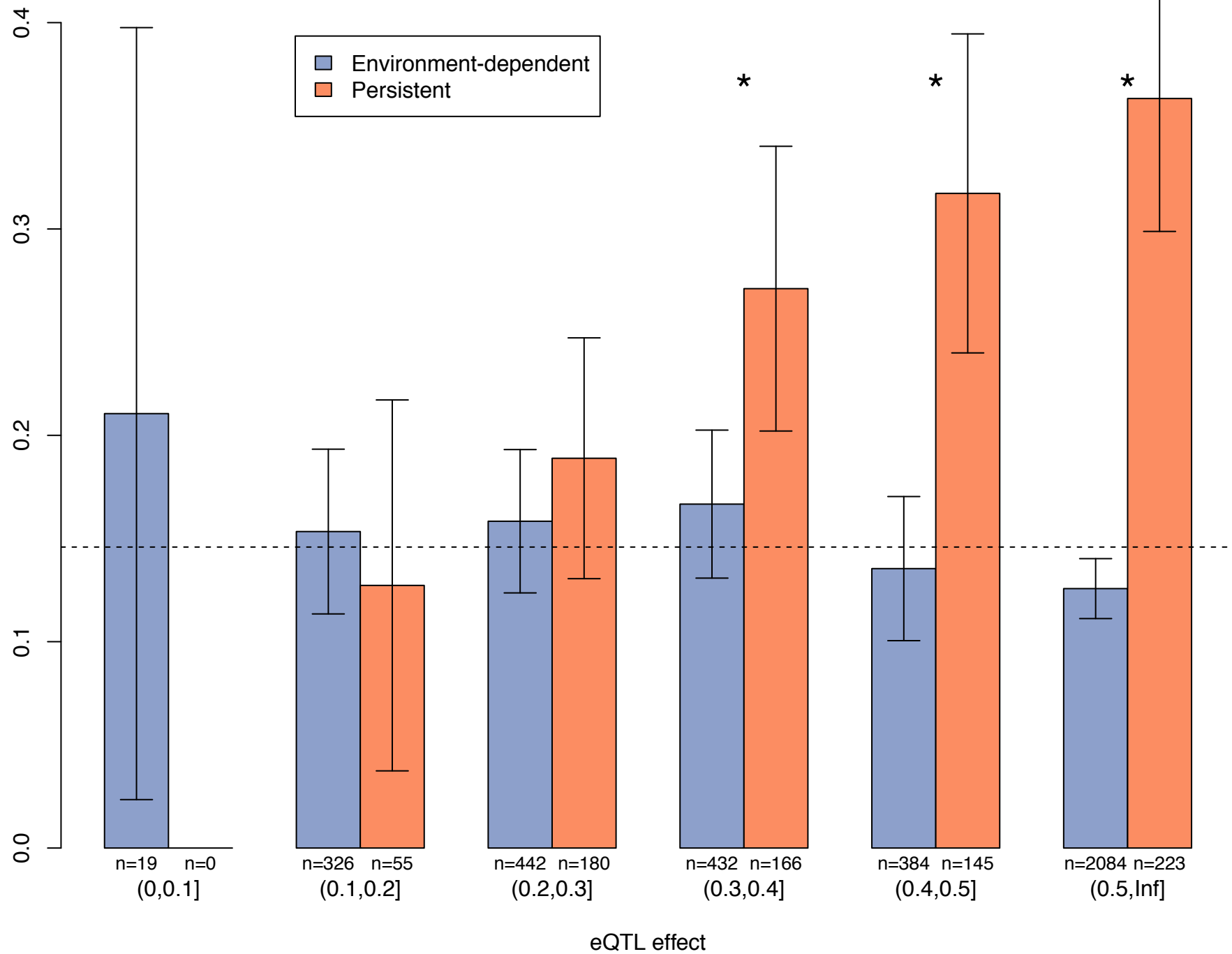

Supplement: Figure S9 — Fraction of validated predictions (y-axis) of candidate mediating genes for environment-dependent eQTLs (blue, FDR<0.05) and environment-persistent eQTLs (orange, FDR<0.05, identified at equivalent sample size, see Text S1) stratified by the eQTL effect (log2 fold change of expression, x-axis). Error bars show two times standard error of the mean, the number of genes in each category is displayed beneath the bar (n = …). Stars indicate significant differences between the two eQTL types (two-sided Fisher test P<0.01) regardless of effect size. (PDF) [file pgen.1003803.s009.pdf]

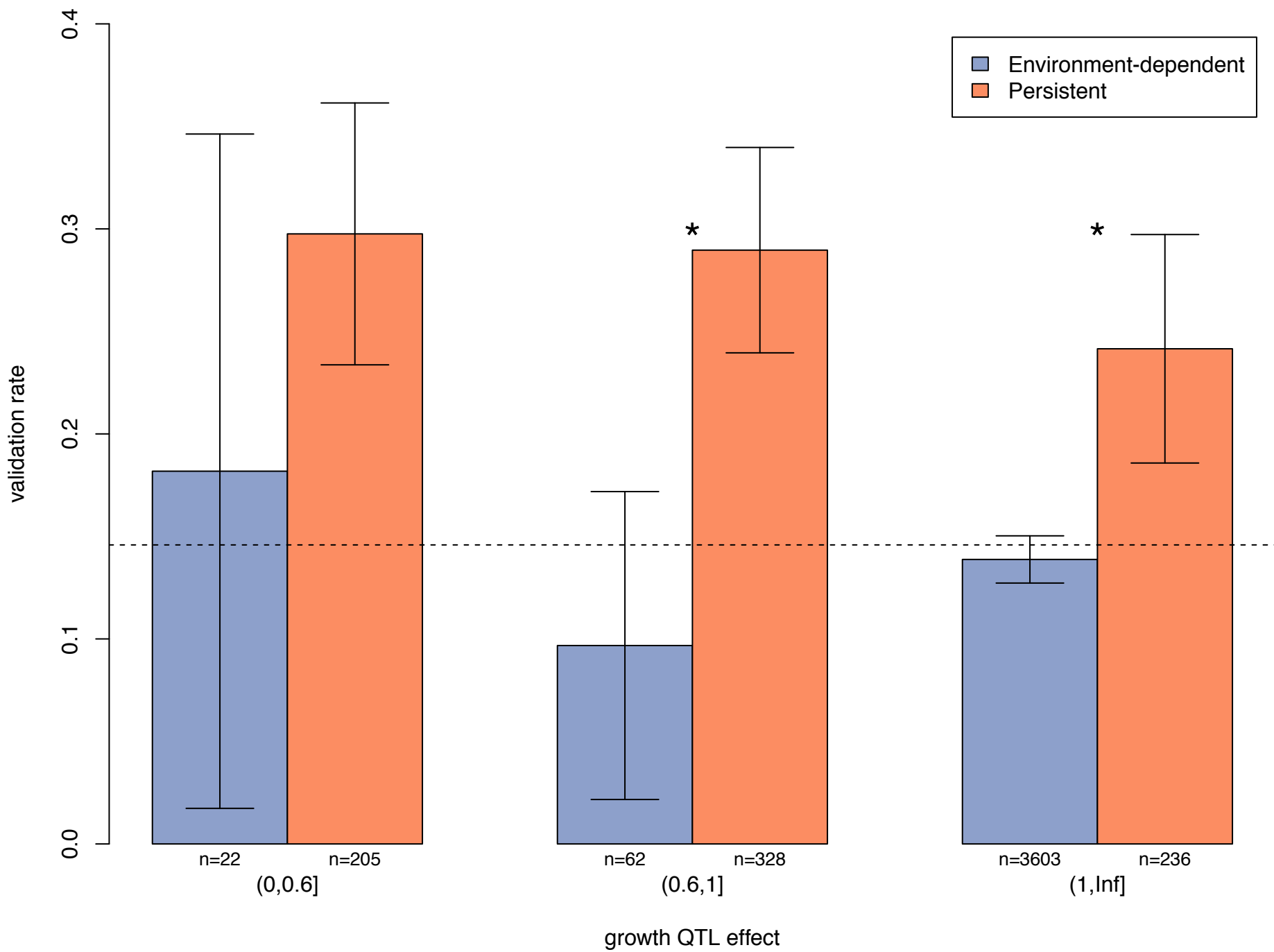

Supplement: Figure S10 — Fraction of validated predictions (y-axis) of candidate mediating genes for environment-dependent eQTLs (blue, FDR<0.05) and environment-persistent eQTLs (orange, FDR<0.05, identified at equivalent sample size, see Text S1) stratified by the growth QTL effect (generations per day, x-axis). The bins have been chosen to contain similar number of QTLs (3, 3 and 4 QTLs respectively). Error bars show two times standard error of the mean, the number of genes in each category is displayed beneath the bar (n = …). Stars indicate significant differences between the two eQTL types (two-sided Fisher test P<0.01) regardless of effect size. (PDF) [file pgen.1003803.s010.pdf]

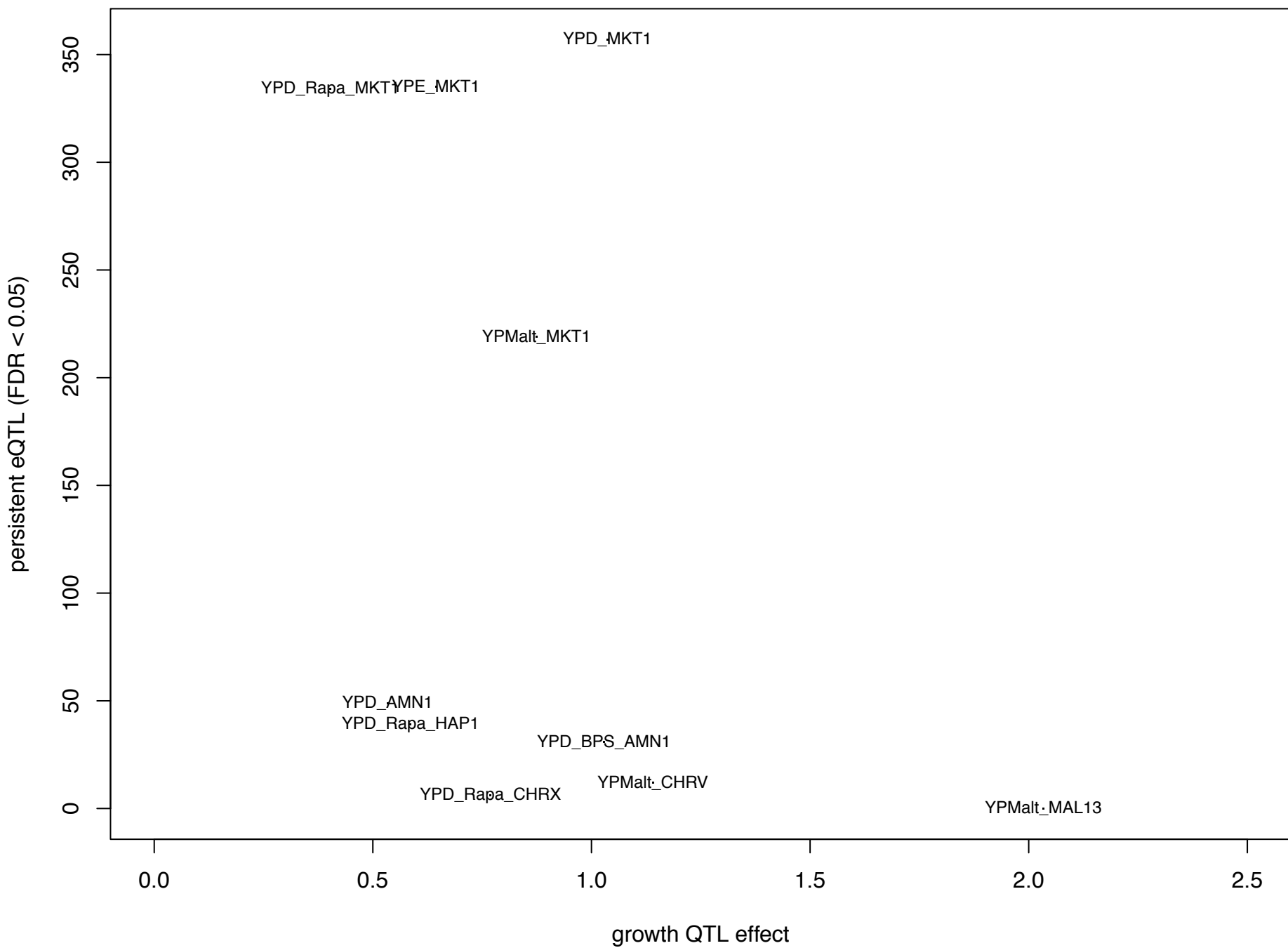

Supplement: Figure S11 — Number of persistent eQTL associations (y-axis, FDR<0.05, identified at equivalent sample size, see Text S1) versus the growth QTL effect (generations per day, x-axis). (PDF) [file pgen.1003803.s011.pdf]

## ***MAL13* validation with RHA**

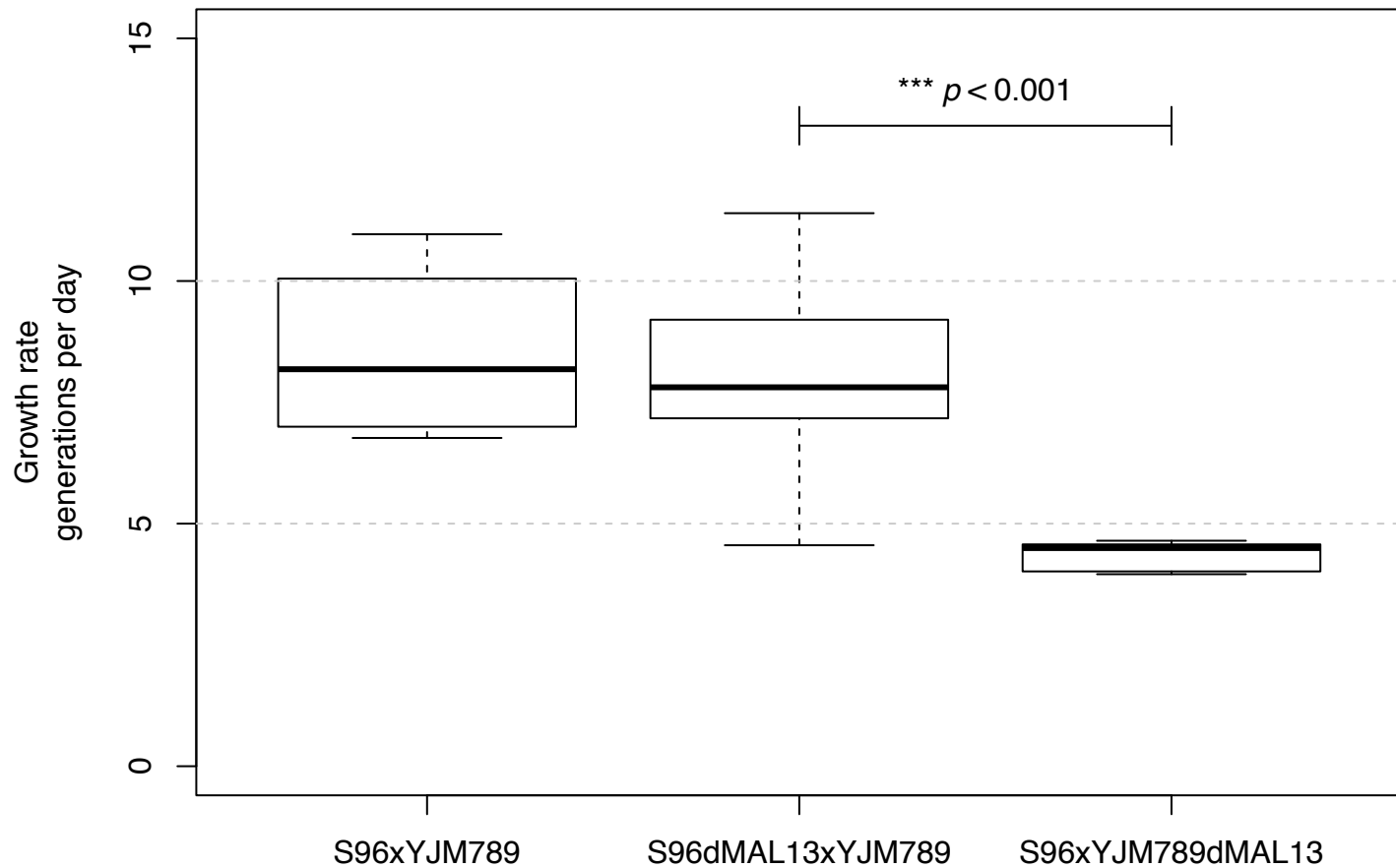

Supplement: Figure S13 — Distribution of growth rate (in generations per day, y-axis) for the hybrid cross between the lab strain and the clinical isolate (S96×YJM789, n = 4), for the hybrid cross where the reference strain allele of MAL13 is deleted (S96dMAL13×YJM789, n = 12) and the hybrid cross where the clinical isolate strain allele of MAL13 is deleted (S96×YJM789dMAL13, n = 6). The latter two differ significantly in growth rate (P<0.001, one-sided Wilcoxon rank sum test). (PDF) [file pgen.1003803.s013.pdf]
