# Supplementary material for: Genotype-Environment Interactions Reveal Causal Pathways That Mediate Genetic Effects on Phenotype
Source: PLoS Genet. 2013 Sep 19;9(9):e1003803. doi: 10.1371/journal.pgen.1003803 (PMC3778020; doi:10.1371/journal.pgen.1003803)
Supplement: Figure S12 — MRP51: example of a candidate gene predicted by the Bayesian network to mediate the effect of MKT1 genotype on growth in ethanol. This corresponds to Figure 3B with a separate y-axis scale for each panel to better show the behavior in the Ethanol environment. In each of the 5 environments (panels), growth rate (y-axis) is plotted vs. expression levels (x-axis) and MKT1 genotype is indicated (clinical isolate allele red, laboratory strain allele blue) for all profiled segregants. MKT1 genotype displays persistent associations with MRP51 expression: the latter segregates with the MKT1 genotype in every environment (vertical bars in each panel mark the midpoint between the expression mean of the two subpopulations). Expression correlates with growth in the Ethanol environment (trend line based on linear regression); MRP51 thereby fulfills all the criteria for a causal intermediate transcript. (PDF) [file pgen.1003803.s012.pdf]

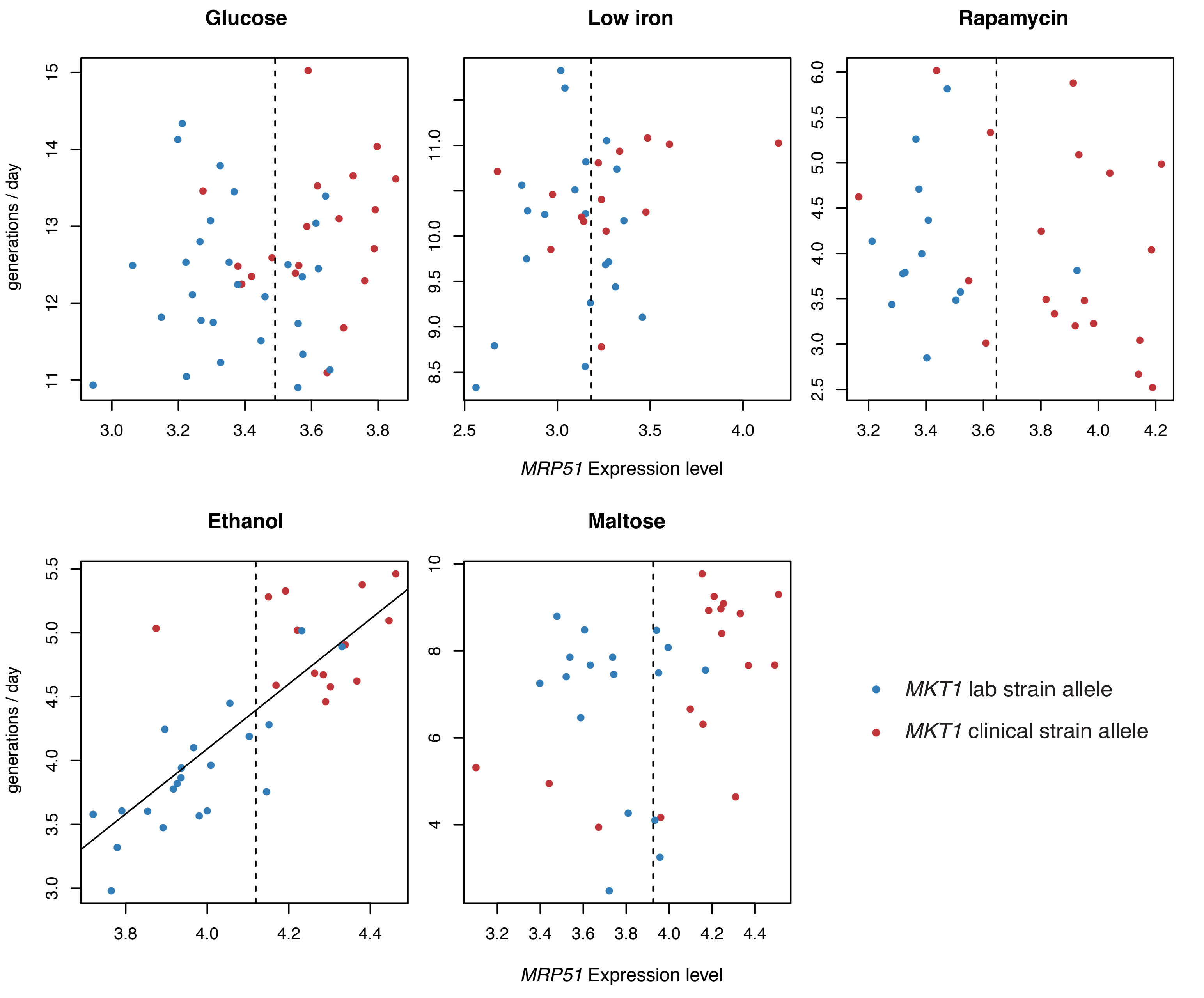

Glucose
Low iron
Rapamycin
Ethanol
Maltose
generations / day
MRP51 Expression level
MKT1 lab strain allele
MKT1 clinical strain allele
