# Supplementary material for: Genotype-Environment Interactions Reveal Causal Pathways That Mediate Genetic Effects on Phenotype
Source: PLoS Genet. 2013 Sep 19;9(9):e1003803. doi: 10.1371/journal.pgen.1003803 (PMC3778020; doi:10.1371/journal.pgen.1003803)
Supplement: Text S1 — Supplementary information. (PDF) [file pgen.1003803.s020.pdf]

# Genotype-environment interactions reveal causal pathways that mediate genetic effects on phenotype
Supplementary Information

Julien Gagneur*, Oliver Stegle*, Chenchen Zhu, Petra Jakob,
Manu M. Tekkedil, Raeka S. Aiyar, Ann-Kathrin Schuon, Dana Pe'er and Lars M. Steinmetz

## Contents

**1 Data availability** **2**

**2 Strains and media** **2**

**3 Growth curves** **2**

**4 Sample preparation for transcription profiling** **2**

**5 Array data analysis** **3**

**6 Transcriptome annotation** **3**

**7 Gene expression levels** **3**

**8 Validation of the *MAL13* growth QTL** **4**

**9 QTL mapping** **4**
9.1 Single-marker single-environment QTL mapping . . . . . 4
9.2 Single-marker interaction tests . . . . . 5
9.3 Counting of QTLs from single marker analysis . . . . . 5
9.4 Multi-environment growth genetic model . . . . . 6

**10 Deletion collection profiling** **6**

**11 Benchmarking** **7**
11.1 Ground truth information and signing of predictions . . . . . 7
11.2 Benchmarking environment-persistent versus environment-dependent associations . . . . . 8
11.2.1 Ranking-based prediction for eQTLs . . . . . 8
11.2.2 Top-ranking 100 predictions per growth QTL and environment . . . . . 8
11.2.3 Overall relative validation rate for 100 top-ranking predictions . . . . . 9
11.2.4 Significant eQTLs, stratified by effect size . . . . . 9

**12 Bayesian network** **9**
12.1 Model comparison . . . . . 9
12.2 Estimating priors on models . . . . . 11
12.3 Posterior and FDR for each model . . . . . 11

13 Pathway enrichment for candidate causal intermediates 12

# 1 Data availability

All tiling array data are accessible at ArrayExpress[1]. The array design is available under the accession number A-AFFY-116.

We used the following genomic DNA hybridizations of Mancera *et al.*[1] (E-TABM-470) for normalization:
recombination_060501_S96, recombination_060501_YJM789,
recombination_060502_S96, recombination_060502_YJM789,
recombination_060503_S96, recombination_060503_YJM789,
recombination_060504_S96, and recombination_060504_YJM789.

We used all cDNA hybridizations of the segregant dataset in YPD media from Xu *et al.*[2] (E-TABM-845). The tiling array data of the cDNA hybridization for all other growth conditions can be downloaded under the accession number E-MTAB-1398.

Moreover, an online archive file at the Steinmetz lab webpage[2] provides large supporting datasets including: raw growth curves, raw data of the deletion collection profile, growth rates and expression levels, score and validation for alternative methods to predict candidate causal intermediate genes.

# 2 Strains and media

The segregant data set consists of 159 of the 184 segregants from Mancera and colleagues[1], derived from a cross of *S. cerevisiae* strains S96 (MATa ho:: lys5 gal2) and YJM789 (MAT$\alpha$ ho::hisG lys2 gal2) (Table S1). Further strains were generated to confirm the *MAL13* QTL (see section 8). The complete list of growth media is given in Table S2.

# 3 Growth curves

Strains were grown and their optical density (OD) were tracked in a TECAN GENios multiwell plate reader. Each plate was done in 2 technical replicates. For the 5 media of focus (those used in transcription profiling), three different plate layouts were assessed in order to minimize well-position effects, leading to a total of 6 growth curves per strain. Growth rates were robustly estimated as the maximum slope of smooth fits of the raw data (local polynomial fit of order 2) using the R/Bioconductor `cellGrowth`[3] package. The bandwidth of the local regression was determined using a ten-fold cross-validation as implemented in the `cellGrowth` package with default parameters. The median value across all replicates was then reported. Explorative analysis showed that these readouts were approximately variance stabilized, and these median raw readings were hence used for all downstream analyses.

# 4 Sample preparation for transcription profiling

The segregants were grown at 30°C to mid-exponential phase ($OD_{600} \simeq 1.0$) in 100 ml YPD_BPS, 100 ml YP_Malt, 200 ml YPE and 400 ml YPD_Rapamycin (see Table S2 for media recipes). Further sample preparation was performed as previously described[2].

[1] http://www.ebi.ac.uk/arrayexpress
[2] http://steinmetzlab.embl.de/gene_env/gagneur_supp_files.tgz
[3] http://www.bioconductor.org/packages/2.11/bioc/html/cellGrowth.html

## 5 Array data analysis

Normalization of tiling array signal was performed as previously described [3, 2]. Briefly, we used the S288c genomic DNA as reference and considered only the probes matching exactly and uniquely to both S288c and YJM789 genomes. We noticed a normalization problem that led to a false eQTL hotspot. To mitigate these effects, we included a quantile normalization step after the variance stabilization step which removed this artifactual association. We estimated background expression levels using the same procedure as previously described [4], based on the intensities of a random set of $10^6$ data points from probes outside transcript boundaries. We set the cutoff for an intensity to be significantly above background at an estimated FDR of 0.05.

## 6 Transcriptome annotation

To obtain the boundaries of all transcribed regions in the dataset, we started with the annotation from the segregants dataset of Xu *et al.*[2]. We manually curated the absent environment-dependent transcribed regions using the Integrative Genomics Viewer (IGV)[4]. To do so, we displayed the expression data in IGV as a heatmap and defined the missing boundaries by using the 'Define a region of interest' function and allowed overlapping transcribed regions on the same strand. Annotated transcribed regions were mapped to open reading frame transcripts (ORF-T) from coding genes defined in the SGD[5] genome annotation (gff file)[6], and to previously annotated regions of Xu *et al.*[5, 2] (SUT for stable unannotated transcript and CUT for cryptic unstable transcript) by reciprocal best hits with at least 80% overlap. Remaining unmapped regions were classified as SUTs. The final transcribed regions were thereafter referred to as *genes*, including both coding and non-coding genes.

## 7 Gene expression levels

Expression levels for non-overlapping genes (section 6) were estimated by taking the median of the normalized probe intensities residing within the gene. For genes overlapping on the same strand, the expression levels were estimated by fitting a model of probe intensities taking each gene's contribution into account. We defined the *transcribed units* as the maximal sets of genes overlapping on the same strand. For a given transcribed unit, let $y_{i,j}$ be the normalized $\log_2$ intensities of probe $i$ in sample $j$, $\beta_{k,j}$ the $\log_2$-expression level of gene $k$ in sample $j$ and let $w_{i,k} = 1$ if probe $i$ overlaps gene $k$ and $w_{i,k} = 0$ otherwise. The $\log_2$ expression levels are then robustly estimated by numerically minimizing the sum of the absolute errors, assuming an additive model in natural (not logarithmic) scale:

$$\hat{\beta} = \arg\min_{\beta} \sum_{i,j} \left| y_{i,j} - \log_2(\sum_k w_{i,k} 2^{\beta_{k,j}}) \right|$$

The estimate is infinite when the expression level is near background. Thus we set a minimal value for the estimates, which corresponds to 5% quantile of $10^5$ random sampled probes below the estimated background level (section 5). Note that in the case of a transcribed unit consisting of a single gene, the solution to the above optimization problem is the median probe intensity. Thus, the two estimation approaches are consistent.

[4]www.broadinstitute.org/igv

[5]Saccharomyces Genome Database: www.yeastgenome.org

[6]updated on 7 March 2007

# 8 Validation of the *MAL13* growth QTL

In order to perform Reciprocal Hemizygote Analysis[6] for the *MAL13* candidate growth QTL, genomic sequence from both strains were required. However, the published genomic sequence for YJM789 lacked the majority of the subtelomeric region of chromosome VII including the MAL1 gene cluster[7]. Therefore, we performed primer walking with Sanger sequencing to determine the sequence between *IMA4* and *PAU12* (primers in Table S6). Reciprocal hemizygote strains for *MAL13* alleles were constructed by crossing relevant YJM789 and S96 background strains (see Table S1).

# 9 QTL mapping

We considered alternative QTL mapping strategies for specific analyses. First, we applied the widely established single-marker analysis method to growth rate and gene expression levels (used in Figure 2 section 9.1). Second, to test for environment-dependent versus environment-persistent gene expression regulation, we employed a linear interaction model (section 9.2). Finally, to investigate and test for causal intermediate transcripts, we developed and applied a stepwise regression approach to define a multi-environment model of growth rate (section 9.4).

Let $p$ be the number of measurements (across segregants and environments) and $N$ the number of genetic markers. For a general phenotypic readout (growth rate or expression of a gene), let $\mathbf{y}$ be the vector of length $p$ of all measurements. When they must be distinguished, we denote the growth rate vector $\mathbf{g}$ and expression of a gene $\mathbf{t}$. Further, let $\mathbf{S} = (\mathbf{s}_1, \ldots, \mathbf{s}_N)$ be the genotype indicator matrix where $\mathbf{S}_{i,j} = 1$ if the segregant profiled in sample $i$ has non-reference allele at marker $j$, 0 otherwise. Let $\mathbf{E} = (\mathbf{e}_1, \ldots, \mathbf{e}_E)$ be the environment indicator matrix, where $\mathbf{E}_{i,j} = 1$ if the $i$th measurement is performed in environment $j$, 0 otherwise.

All QTL mapping analyses were carried out either on the growth data or the expression data. Note that due to the sparse expression profiling (see Fig. S1), the vectors $\mathbf{t}$ contain missing values. In the likelihood calculation, rows with missing values are dealt with explicitly, which for most models is equivalent to restricting the set of measurements to those segregants that have been transcription profiled in the corresponding environments.

**False Discovery Rate** For each phenotype individually, we estimated False Discovery Rate (FDR) using the $q$-value package[8].

## 9.1 Single-marker single-environment QTL mapping

Single marker mapping was carried out using a standard linear association model with a Gaussian observation noise:

$$p(\mathbf{y}_e \,|\, \theta, \beta_0, \sigma^2) = \mathcal{N}\left(\mathbf{y}_e \,\middle|\, \underbrace{\theta \mathbf{s}_e}_{\text{genetic effect}} + \underbrace{\beta_0 \mathbf{1}}_{\text{bias term}}, \sigma^2 \mathbf{I}\right). \quad (1)$$

Here, $\mathbf{y}_e$ is the phenotype restricting the observations to a particular environment $e$, ignoring all others. Similarly, $\mathbf{s}_e$ denotes the matching genotype information of those segregants that have been phenotyped in environment $e$. Significance of the genetic effect $\theta$ is then assessed using likelihood ratio tests, and $\beta_0$ denotes the weight for the bias term. Note that for single-environment tests, there is no need to account for structure within the population, as individual segregants are measured at most once in each environment and hence all samples can be considered as unrelated. In joint association tests, relating multiple environments, this assumption no longer holds and hence we employed a mixed model approach to account for the relatedness of samples; see section 9.2 and section 9.4.

### 9.2 Single-marker interaction tests

To test for environment-dependent versus environment-persistent genetic effects, we fitted a model across all environments, assuming an effect size shared across environments as well as a specific term:

$$\begin{pmatrix}\mathbf{y}_1\\ \vdots\\ \mathbf{y}_i\\ \vdots\\ \mathbf{y}_E\end{pmatrix} = \underbrace{\theta_0\begin{pmatrix}\mathbf{s}_n\\ \vdots\\ \mathbf{s}_n\\ \vdots\\ \mathbf{s}_n\end{pmatrix}}_{\text{main effect}} + \underbrace{\theta_i\begin{pmatrix}\mathbf{0}\\ \vdots\\ \mathbf{s}_i\\ \vdots\\ \mathbf{0}\end{pmatrix}}_{\text{specific effect for environment } i} + \underbrace{\begin{pmatrix}\beta_1\mathbf{I}\\ \vdots\\ \beta_i\mathbf{I}\\ \vdots\\ \beta_E\mathbf{I}\end{pmatrix}}_{\text{bias terms}} + \underbrace{\mathbf{u}}_{\text{population structure random effect}} + \underbrace{\begin{pmatrix}\boldsymbol{\psi}_1\\ \vdots\\ \boldsymbol{\psi}_i\\ \vdots\\ \boldsymbol{\psi}_E\end{pmatrix}}_{\text{noise term}}. \quad (2)$$

Here, we split the phenotype $\mathbf{y} = (\mathbf{y}_1, \ldots, \mathbf{y}_i, \ldots, \mathbf{y}_E)$ into profiling information in the considered environments $e = 1 \ldots E$.

Because identical segregants have been phenotyped in different environments, the individual samples in Equation (2) are no longer i.i.d. To account for this replicate sample structure, we include a random effect term $\mathbf{u} \sim \mathcal{N}(\mathbf{0}, \mathbf{K})$, where $\mathbf{K}$ is solely estimated from the replicate structure, i.e.

$$\mathbf{K}_{i,j} = \begin{cases} 1 & \text{if sample } i \text{ and } j \text{ are identical genotypes} \\ 0 & \text{otherwise} \end{cases}.$$

Assuming a distinct variance parameter $\sigma_e^2$ for each of the noise terms, i.e. $\boldsymbol{\psi}_e \sim \mathcal{N}(0, \sigma_e^2)$, the likelihood that correspond to the model implied by Equation (2) is

$$p(\mathbf{Y} \mid \theta_0, \theta_i, \beta_1, \ldots, \beta_E, \mathbf{K}, \sigma^2, \delta_1^2, \ldots, \delta_E^2) = \quad (3)$$
$$\mathcal{N}\left(\begin{pmatrix}\mathbf{y}_1\\ \vdots\\ \mathbf{y}_i\\ \vdots\\ \mathbf{y}_E\end{pmatrix} \middle| \underbrace{\theta_0\begin{pmatrix}\mathbf{s}_n\\ \vdots\\ \mathbf{s}_n\\ \vdots\\ \mathbf{s}_n\end{pmatrix}}_{\text{genetic main effect}} + \underbrace{\theta_i\begin{pmatrix}\mathbf{0}\\ \vdots\\ \mathbf{s}_i\\ \vdots\\ \mathbf{0}\end{pmatrix}}_{\text{interaction effect}} + \underbrace{\begin{pmatrix}\beta_1\mathbf{I}\\ \vdots\\ \beta_i\mathbf{I}\\ \vdots\\ \beta_E\mathbf{I}\end{pmatrix}}_{\text{environment effect}}, \sigma^2\left(\mathbf{K} + \begin{bmatrix}\delta_1^2\mathbf{I} & & & & \\ & \ddots & & & \\ & & \delta_i^2\mathbf{I} & & \\ & & & \ddots & \\ & & & & \delta_E^2\mathbf{I}\end{bmatrix}\right)\right).$$

Again, we employ likelihood ratio tests to assess significance of either the main effect ($\theta_0$) or the interaction term ($\theta_i$). To speed up the computation, we fit the relative noise levels $\delta_1^2, \ldots, \delta_E^2$ on the null model, ignoring the genetic effect. During testing on genome-wide markers, only the absolute scale of the covariance ($\sigma^2$) is refit for every genetic variant. This approximation is analogous to the approach used in EMMA-X[9] and is most accurate for small genetic effect sizes. In the context of multiple trait models, this approximation has also been considered in [10].

### 9.3 Counting of QTLs from single marker analysis

Except when otherwise stated, the reported results were obtained from a single marker QTL mapping. The number of QTLs are given under the assumption of at most one QTL per chromosome and phenotype to avoid confounding the number of true regulatory effects with extended linkage blocks. The joint mapping, as done in the multi-environment growth genetic model, avoids the need for such measures, as individual interaction terms are fitted conditioning on all others; see Section 9.4 for details.

### 9.4 Multi-environment growth genetic model

We employed a stepwise regression model to construct a global growth genetic model across all environments and relevant loci jointly. In this fitted model, a marker with a non-zero effect size in a particular environment is denoted a genotype-environment interaction term. Each such interaction is defined by locus index $n_i$ and an environment index $e_i$, where growth is affected.

**Joint likelihood model** Let us first assume the set of $N_t$ interaction terms was known. Conditioned on this set of loci and environment indicators $(n_i, e_i)_{i=1}^{Nt}$, the likelihood of growth rate can be written as

$$p(\mathbf{g} \,|\, \mathbf{E}, \boldsymbol{\beta}, \mathbf{S}, (n_i, e_i)_{i=1}^{Nt}, \boldsymbol{\theta}, \mathbf{K}, \sigma^2, \boldsymbol{\delta}) = \tag{4}$$
$$\mathcal{N}\left(\mathbf{g} \,\middle|\, \mathbf{E}\boldsymbol{\beta} + \sum_{i=1}^{Nt} \theta_i \delta_{(\mathbf{e}==e_i)} \odot \mathbf{s}_{n_i}, \sigma^2 \left(\mathbf{K} + \begin{bmatrix} \delta_1^2\mathbf{I} & & & & \\ & \ddots & & & \\ & & \delta_i^2\mathbf{I} & & \\ & & & \ddots & \\ & & & & \delta_E^2\mathbf{I} \end{bmatrix}\right)\right).$$

Here, $\mathbf{E}\boldsymbol{\beta}$ denotes a vectorized representation of the environment-dependent bias term in Equation (3). Similarly, we have defined $\boldsymbol{\beta} = (\beta_1, \ldots, \beta_E)$ and $\boldsymbol{\delta} = (\delta_1, \ldots, \delta_E)$. Just as introduced for the single marker interaction tests (section 9.2), the covariance structure is included to explain the relatedness when identical genotypes are profiled in several environments. The variable $\delta_{\mathbf{e}==e_i}$ is the indicator variable for environment $e_i$. This model is closely related to recently proposed multiple trait mixed models[10].

**Fitting of interactions** To fit the model implied by Equation (4), we employ a stepwise procedure, greedily including terms to the set of interaction when starting from an empty model ($N_t = 0$). Genome-wide significant genotype-environment effects are then added to the current set of interactions. This iterative learning approach is repeated until no additional marker reaches genome-wide significance (FDR cutoff, see Section 9). As done in [10], the relative noise levels $\delta_1, \ldots, \delta_E$ are updated in every iteration and held constant for genome-wide scans.

At an FDR of 5%, this approach resulted in 6 unique loci that had a non-zero effect size in 10 combinations of environments and markers. The position of markers with an effect are also illustrated in Figure 2B.

**Naming of growth QTLs** Among the identified 6 unique loci of the multi-environment growth genetic model, 3 of them were in close vicinity to previously validated QTLs in crosses of these backgrounds (ref. [6] for *MKT1*) or in a cross that also involved the lab reference strain (ref. [11] for *HAP1* and ref. [12] for *AMN1*) and were named after the identified genetic loci. We identified and validated *MAL13* (section 8). We named the remaining 2 growth QTLs after the chromosome number in which they lie (CHRV and CHRX).

## 10 Deletion collection profiling

Aliquots of the deletion collection were obtained from Robert St. Onge (Stanford Genome Technology Center, Palo Alto, CA). We expanded the original deletion collection pool by growing the cells in YPD overnight at 30°C and prepared glycerol stocks (15% glycerol). For the experiment, we thawed triplicates in each growth condition in 50 mL of media and collected the cells after 5 generations. Genomic DNA extraction, PCR amplification of molecular tags, and Genflex tag16k array (Affymetrix) hybridization, washing, and scanning were performed as previously described[13]. The raw microarray data are available in the file deletion_collection_raw.zip in online archive (Section 1).

Each probe on the Genflex tag16k array (Affymetrix) is represented by 5 replicate features. Each tag was summarized by the $\log_2$-median intensity across all matching probes on the array. We then adjusted for overall signal intensity and PCR amplification of up and down tags as follows. For each pool, some tags do not yield a signal above background levels, either because the strain has completely vanished from the pool, or it does not grow, or because the tag has acquired too many mutations and therefore does not hybridize to the array. Only $\log_2$ intensities above 7 (a cutoff set from visual inspection of the intensity distributions as separating a low from a high intensity sub-population) were considered above background. The $\log_2$ intensity distributions of the up and down tag populations in each microarray were shifted by a separate constant so that all intensity distributions have the same midpoint of the shortest interval containing half the data (a robust estimator of the mode of a distribution).

For each strain, the selection coefficient $s$ (i.e. the relative growth rate compared to the pool) was estimated using a linear model of the $\log_2$ intensities $y_{i,j}$ that takes the identity of the tag as an additive covariate to control for differences in hybridization efficiency: $y_{i,j} = \beta_0 + \beta_{up} u_j + s g_i + \psi_{i,j}$

where:

- $u_j$ values 1 if the tag j is the uptag, 0 otherwise
- $g_i$ is the generation number
- $\beta_0, \beta_{up}$ are intercept and relative affinity of the uptag
- $\psi_{i,j}$ is noise

Significance for the selection coefficient was determined using the moderated t-test implemented in the R/Bioconductor `limma` package. P-values were corrected for multiple testing with the Benjamini-Hochberg method. In our setup, many strains with slow growth rates on rich media are depleted and already not detectable at the initial time points. Previously, Steinmetz and colleagues[14] have let the slower growing strains grow on plates longer so that they enter the pool at similar initial amounts as the other strains. For the two media available (YPD and YPE), we have used estimates obtained by reanalyzing the original dataset[14] with our analysis pipeline.

# 11 Benchmarking

Several alternative benchmarks and comparisons were considered. First, Section 11.1 describes how the ground truth information was obtained from the deletion data and how predictions from individual methods can be "signed" to distinguish detrimental from beneficial effects. Section 11.2 describes the analysis of environment-dependent versus environment-persistent eQTLs in detail, including a number of alternative analysis approaches.

## 11.1 Ground truth information and signing of predictions

**Matching genes from the transcriptome annotation and deletion strains** The deletion collection we used targets non-essential coding genes. Genes annotated as an ORF-T (see Section 6)) were first matched to the 0, 1 or many strains of the deletion collection for the same ORF as described by Pierce and colleagues[13]. Non-coding genes (SUTs) were considered as not having any matching deletion strains. For each gene in each media, the FDR and selection coefficients (section 10) across all possible matched strains of a gene were summarized by the median. Altogether, the matching of genes to deletion strains led to 4,498 distinct non-essential genes from a total of 5,230 pooled DNA-barcoded strains.

**Ground truth information for beneficial and detrimental effects** In each media, two separate predictions were considered: predictions for growth improvement on the one hand and predictions for growth impairment on the other hand. Genes were classified as *improved growth upon deletion* in one media if the effect was significant (FDR $< 0.05$) and positively large ($s > 0.05$), and were not classified as such otherwise.

Similarly, genes were classified as *impaired growth upon deletion* in one media if the effect was significant (FDR $< 0.05$) and negatively large ($s < -0.05$), and were not classified as such otherwise.

**Signed predictions** Alternative methods (see section 11.2) were considered to predict detrimental or beneficial effects on the growth phenotype. To score these predictions, we consider a signed scoring approach, accounting for the correlation between gene expression and growth to identify the directionality of the effect. Each method $m$ (environment-persistent eQTL association, environment-dependent, Bayesian network, etc.) that predicts a gene $i$ to mediate the effect of a combination of one growth QTL $j$ in a relevant media $k$ returns a significance as $\mathrm{FDR}_{m,i,j,k}$.

For the task of predicting growth impairment upon deletion, we defined the *score* $T^1_{m,i,j}$ as

$$T^1_{m,i,j,k} = -\frac{\rho(G_k, Y_{i,k})}{|\rho(G_k, Y_{i,k})|} \log_{10}(\mathrm{FDR}_{m,i,j,k}) \tag{5}$$

$$\tag{6}$$

where $\rho()$ is the Spearman rank correlation between the growth rate $G$ in environment $k$, and the expression $Y$ of gene $i$ in environment $k$. For the task of predicting growth improvement upon deletion, we defined the score as the opposite value of the former: $T^{-1}_{m,i,j} = -T^1_{m,i,j}$. For each prediction task, the higher the score is, the stronger the prediction.

## 11.2 Benchmarking environment-persistent versus environment-dependent associations

Several alternative benchmarking approaches were considered to ensure that the enrichment for validated genes among the environment-persistent associations was robust. All eQTL mapping was carried out in a focused fashion at the set of the 6 growth QTLs with an effect in 10 genotype/environment combinations as obtained from the multi-environment growth genetic model (see Section 9.4). In particular, this alters the correction for multiple testing of eQTL mapping, as only specific hypotheses are tested rather than carrying out genome-wide association mapping.

To rule out possible biases due to sample size, environment-persistent eQTLs were also assessed using a subsampled dataset, such that the number of data points matched the average number of measurements in any specific environment. However, operating on the full data give very similar results, suggesting that sample size is not a main determinant of the observed differences (Fig. S7, see section 11.2.1).

### 11.2.1 Ranking-based prediction for eQTLs

First, we considered a ranking-based approach jointly across all 6 growth loci in the relevant environments (see multi-environment growth genetic model, Section 9.4). Fig. S7 shows the validation rate of employing either environment-dependent or environment-dependent eQTL mapping as a function of the rank cutoff to call the respective categories of association. These results are contrasted with a random selection approach, which is expected to have a validation rate equal to the overall genome-wide rate of impaired (respectively improved) deletion phenotypes. Because these rates depend on the environment considered (Fig. S3), we considered the *relative validation rate* in each environment as the rate of validation divided by the genome-wide validation rate.

### 11.2.2 Top-ranking 100 predictions per growth QTL and environment

Second, we considered the top-ranking 100 associations for each growth QTL and environment, which ensures that the number of predictions from each growth locus and environment is equal and not dominated by a small number of loci (such as *MAL13* with $> 2,000$ environment-dependent eQTLs). Fig. S8 depicts the enrichment of these top ranking 100 environment-dependent versus environment-persistent eQTLs at each locus and environment-dependent eQTLs, compared with a random predictor as before.

### 11.2.3 Overall relative validation rate for 100 top-ranking predictions

Third, we provide the overall validation rate across the top 100 ranking predictions, combining the evidence from all 6 loci in the relevant environments. This genome-wide summary of validation rate for every growth QTLs was robustly estimated using a jack-knife resampling scheme (main paper, Figure 3A). Overall, the relative validation rate for environment-persistent and environment-dependent associations was computed 10 times, each time removing data for one of the 10 relevant combination of growth QTL and environment. This resampling procedure yielded 10 paired estimates of the overall relative validation rates. Significance of difference was computed using the two-sided paired Wilcoxon rank sum test.

### 11.2.4 Significant eQTLs, stratified by effect size

Finally, we considered the impact of effect sizes of environment-dependent and environment-persistent eQTLs. We used this additional control to ensure that the improved prediction from environment-persistent eQTLs is not simply caused by larger effect sizes of this association category. Fig. S9 shows the overall validation rate across all 6 loci in the relative environments in different effect size categories. In each bin, genes were selected by effect size and filtered for significance (FDR $< 0.05$). The effect size distribution from persistent and dependent eQTLs was similar (total number of eQTLs in each category), with persistent eQTLs being validated at a higher rate, independent of effect size. As expected, the difference between the two association categories was most pronounced for eQTLs with larger effect sizes.

# 12 Bayesian network

To predict causal intermediates, we first fit a joint genetic growth model (section 9.4). Next, for each of the 10 interaction terms in the model and for each gene, we use model comparison to predict the role of individual genes for each such term.

As introduced in section 9, let $\mathbf{g}$ be the $p$-vector of the growth rates in all $p$ samples, $\mathbf{t}$ denote the $p$-vector of gene expression a gene of interest, $\mathbf{s}$ be the $p \times N$ genotype indicator matrix and $\mathbf{E}$ the $p \times 5$ environment indicator matrix. As the modeling considered here relates gene expression to growth rate, samples where gene expression has not been profiled are treated as missing data.

**Data normalization** To be robust against outliers and possible non-linearities, we transformed phenotype $y$ (gene expression or growth rate) into a rank-standardized form $\tilde{y}$ following [15]:

$$\tilde{y_k} = \mathbf{\Phi}^{-1}\left(\frac{\text{rank}(y_k)}{(n+1)}\right), k = 1, \ldots, n. \tag{7}$$

where $\mathbf{\Phi}$ denotes the cumulative distribution function of a normal distribution. This transformation projects rank data to unit variance Gaussian. The rank normalization was carried out in every environment independently, as the direct environmental effect is not part of the scoring scheme and explained away by bias terms for each environment. For comparison, we also considered the analogous models using original values for gene expression and growth rates. While the general conclusions were unaltered, the rank scale appeared to be more robust (see also Chen et al. [15] for a discussion).

## 12.1 Model comparison

Alternative hypotheses for the mediating role of genes are encoded in alternative marginal likelihood models for growth rate and gene expression levels. To assess the fit of these models to data, we employed marginal likelihood-based scoring, which is similar to the likelihood-ratio based test for Mendelian randomization used elsewhere (e.g. [16, 17]).

Each interaction term of the genetic model is defined by a marker index $n_i$ and the index of the relevant environment $e_i$. Note that if markers affect growth in multiple environments, the identical marker index will

appear multiple times in the converged growth model; the individual genotype-environment interactions of the fitted multi-environment model are visualized in the main text, Figure 1.

**Model $\mathcal{M}_1$: Gene expression mediates the genotype-environment interaction** If expression of a gene mediates a specific interaction of the growth genetic model, then growth rate and genotype at the interaction marker are independently conditioned on gene expression. Furthermore, the gene is under environmentally persistent regulation of the corresponding marker. For a particular interaction term $(n_i, e_i)$, the joint distribution encoding these statistical dependencies is

$$p(\mathbf{g}, \mathbf{t} \,|\, \mathbf{E}, \mathbf{s}_{n_i}, e_i) = \underbrace{p(\mathbf{g} \,|\, \mathbf{E}, \mathbf{t}, e_i)}_{\text{growth model}} \; \underbrace{p(\mathbf{t} \,|\, \mathbf{E}, \mathbf{s}_{n_i})}_{\text{expression model}} \; . \tag{8}$$

Here, the growth model is identical to the multi-environment growth genetic model, where the marker of the specific genotype-environment interaction in question is substituted with the gene expression profile $\mathbf{t}$:

$$p(\mathbf{g} \,|\, \mathbf{S}, \mathbf{E}, \boldsymbol{\beta}, \mathbf{t}, (n_i, e_i), \theta_i, \sigma^2) = \mathcal{N}\left(\mathbf{g} \,\middle|\, \mathbf{E}\boldsymbol{\beta} + \mathbf{v} + \theta_i \delta_{\mathbf{e}==e_i} \odot \mathbf{t}, \sigma^2 \mathbf{I}\right). \tag{9}$$

where $\mathbf{v} = \sum_{j \neq i} \theta_j \delta_{(\mathbf{e}==e_j)} \odot \mathbf{s}_{n_j}$, denotes the remaining $N_t - 1$ factors contributing to the growh model but the term being tested (See section 9.4).

We place Gaussian priors on all weights

$$p(\boldsymbol{\beta}) = \prod_{e=1}^{E} \mathcal{N}\left(\beta_e \,\middle|\, 0, \sigma_\beta^2\right) \tag{10}$$

$$p(\boldsymbol{\theta}) = \prod_{i=1}^{N} \mathcal{N}\left(\theta_i \,\middle|\, 0, \sigma_{\theta_i}^2\right), \tag{11}$$

where we assume that the variance contribution of individual environments is shared whereas the genetic terms have specific variance parameters.

The gene expression model is designed correspondingly, where the marker effect acts equally across all environments, modeling environmental persistence:

$$p(\mathbf{t} \,|\, \mathbf{S}, \mathbf{E}, \boldsymbol{\beta}, \theta, n_i, \sigma^2) = \mathcal{N}\left(\mathbf{t} \,\middle|\, \mathbf{E}\boldsymbol{\beta} + \theta \mathbf{s}_{n_i}, \sigma^2 \mathbf{I}\right). \tag{12}$$

Prior distributions for environmental weights and the marker effect are chosen in an identical manner

$$p(\boldsymbol{\beta}) = \prod_{e=1}^{E} \mathcal{N}\left(\beta_e \,\middle|\, 0, \sigma_\beta^2\right) \tag{13}$$

$$p(\theta) = \mathcal{N}\left(\theta \,\middle|\, 0, \sigma_\theta^2\right). \tag{14}$$

The marginal likelihood of the joint probability distribution in Equation (8) is estimated using a likelihood type II approach, marginalizing over the weight parameters $\boldsymbol{\beta}$, $\boldsymbol{\theta}$ and optimizing over the hyperparameters $\sigma_\beta^2$, $\sigma_{\theta_i}^2$ and $\sigma^2$.

**Model $\mathcal{M}_0$: Gene expression is independent of the genotype-environment interaction** The null model, in which gene expression variability is unrelated to the genotype to growth rate association, is constructed in an analogous fashion.

$$p(\mathbf{g}, \mathbf{t} \,|\, \mathbf{E}, \mathbf{s}_{n_i}, e_i) = \underbrace{p(\mathbf{g} \,|\, \mathbf{E}, \mathbf{s}_{n_i}, e_i)}_{\text{growth model}} \quad \underbrace{p(\mathbf{t} \,|\, \mathbf{E})}_{\text{expression model}} \quad . \tag{15}$$

Here, the growth model directly corresponds to the multi-environment growth genetic model

$$p(\mathbf{g}\,|\,\mathbf{S},\mathbf{E},\boldsymbol{\beta},(n_i,e_i),\theta_i,\sigma^2) = \mathcal{N}\left(\mathbf{g}\,\middle|\,\mathbf{E}\boldsymbol{\beta}+\mathbf{v}+\theta_i\delta_{\mathbf{e}==e_i}\odot\mathbf{s}_{n_i},\sigma^2\mathbf{I}\right), \tag{16}$$

and the gene expression model assumes the gene is not under genetic regulation

$$p(\mathbf{t}\,|\,\mathbf{E},\boldsymbol{\beta},\sigma^2) = \mathcal{N}\left(\mathbf{t}\,\middle|\,\mathbf{E}\boldsymbol{\beta},\sigma^2\mathbf{I}\right). \tag{17}$$

The prior distributions on the respective weight parameters and the marginalization are identical to the approach taken for Model 1.

**Approximate model posterior using BIC** Next, model $\mathcal{M}_0$ and model $\mathcal{M}_1$ were compared, controlling for the different number of parameters. Building on the marginal likelihood of the data under each model, we derived Bayesian Information Criterion (BIC) scores to approximate exact Bayes factors that would involve marginalizing out the remaining model parameters as well. The BIC correction is defined as follows

$$\ln p(\mathbf{t},\mathbf{g}\,|\,\mathcal{M}_i) \approx \underbrace{\ln p(\mathbf{t},\mathbf{g}\,|\,M_i,\boldsymbol{\theta}) - 0.5\ln|\boldsymbol{\theta}|\ln(p)}_{\ln\tilde{p}(\mathbf{t},\mathbf{g}\,|\,\mathcal{M}_i)}, \tag{18}$$

where $\ln p(\mathbf{t},\mathbf{g}\,|\,\mathcal{M}_i,\boldsymbol{\theta})$ denotes the marginal likelihood term under model $i$ that still depends on parameters $\boldsymbol{\theta}$ and $p$ denotes the number of samples.

## 12.2 Estimating priors on models

Using approximate marginal likelihoods (Equation (18)) for every gene and both models, priors for the two alternative models ($p(\mathcal{M}_0) = \pi_0$ and $p(\mathcal{M}_1) = 1-\pi_0$) were estimated. This was done by numerically maximizing the genome-wide (*i.e.* across all genes) likelihood:

$$p(g,\mathbf{T}|\pi_0) = \prod_i p(g,T_i|\pi_0) \tag{19}$$

$$= \prod_i (p(g,T_i|\mathcal{M}_0)\pi_0 + p(g,T_i|\mathcal{M}_1)(1-\pi_0)) \tag{20}$$

$$\tag{21}$$

where $\mathbf{T}$ is the matrix of all gene expression levels and $T_i$ its columns (single gene expression vector). Note that $p(g,\mathbf{T}|\pi_0)$ is a polynomial in $\pi_0$ which has a single local maximum in $[0,1]$ for any positive values of $p(g,T_i|\mathcal{M}_0)$ and $p(g,T_i|\mathcal{M}_1)$. This can be shown by noticing that all roots are real and outside $[0,1]$. Hence convergence to the global optimum with local optimization techniques is ensured.

## 12.3 Posterior and FDR for each model

Finally, the approximated posterior probability for model $\mathcal{M}_1$ reads

$$\tilde{p}(\mathcal{M}_1\,|\,\mathbf{g},\mathbf{s},\mathbf{E},\mathbf{t}) = \frac{\tilde{p}(\mathbf{t},\mathbf{g}\,|\,\mathcal{M}_1)(1-\pi_0)}{\tilde{p}(\mathbf{t},\mathbf{g}\,|\,\mathcal{M}_1)(1-\pi_0)+\tilde{p}(\mathbf{t},\mathbf{g}\,|\,\mathcal{M}_0)\pi_0}. \tag{22}$$

To compare results with other FDR-based methods, false discovery rates were derived from the posterior for model $\mathcal{M}_1$.

For a statistic $T$ that decreases with significance, the expected FDR for a cutoff value $c$ is $P(\mathcal{M}_0|T \leq c)$. We considered as statistic for gene $i$ the estimate for the posterior of model $\mathcal{M}_0$, $\tilde{p_i^0} = \tilde{p}(\mathcal{M}_0\,|\,\mathbf{g},\mathbf{s},\mathbf{E},\mathbf{t}_i)$.

Let $\mathcal{S}_i = \left\{ j | \tilde{p_j^0} \leq \tilde{p_i^0} \right\}$ the set of genes with an estimated posterior for $\mathcal{M}_0$ less or equal to the estimated posterior for $\mathcal{M}_0$ of gene $i$. We estimated the FDR for gene $i$ as

$$\mathrm{FDR}_i \simeq P(\mathcal{M}_0 | \tilde{p}^0 \leq \tilde{p}_i^0) \quad (23)$$

$$\mathrm{FDR}_i \simeq \frac{\sum_{j \in \mathcal{S}_i} \tilde{p}_j^0}{\#\mathcal{S}_i} \quad (24)$$

Related Bayesian interpretations of q-values and FDR cutoffs are discussed in [18].

# 13 Pathway enrichment for candidate causal intermediates

We calculated pathway enrichments for the predicted causal intermediate genes (Bayesian Network, Section 12) at each genotype-environment interaction of the growth genetic model (Section 9.4). For this analysis, to obtain a sufficiently large set of predictions for enrichment analysis, we considered an FDR cutoff (FDR $< 0.05$), yielding 3,680 total predictions across all 10 combinations of growth QTL and corresponding environments.

**Statistical testing and identified categories** For the predictions at each locus and environment, we calculated enrichment for Gene Ontology, KEGG pathways and regulatory target sites separately. Significant enrichments were assessed using Fisher's exact test, where we corrected for the number of enrichment terms tested with the Benjamini-Hochberg correction. Enrichments with a significance level of FDR $< 0.1$ are provided as Table S4.

Gene Ontology annotations were obtained from the SGD (`http://www.yeastgenome.org`) on 7 March 2007. The data for regulatory target network is from MacIsaac *et al.* [19]. The data for RNA binding proteins is from Hogan *et al.* [20].

**Result summary** Downstream of the majority of the loci of the growth genetic model, we predicted mediating genes that were enriched for GO-categories with plausible functions. In particular we found mitochondrial genes associated with *MKT1* in all 4 media, targets of the transcription factor Hap1 among candidate causal intermediates for the *HAP1* QTL, and cytokinetic cell separation for the *AMN1* QTL, a protein required for daughter cell separation. As expected from the model that searches for persistent mediating genes, no difference in functional enrichment was observed between the different environments, even though the exact predicted genes differed.

# References


[1] Mancera, E., Bourgon, R., Brozzi, A., Huber, W. & L., S. High-resolution mapping of meiotic crossovers and non-crossovers in yeast. *Nature* (2008).

[2] Xu, Z. *et al.* Antisense expression increases gene expression variability and locus interdependency. *Molecular Systems Biology* **7** (2011).

[3] Huber, W., Toedling, J. & Steinmetz, L. M. Transcript mapping with high-density oligonucleotide tiling arrays. *Bioinformatics (Oxford, England)* **22**, 1963–1970 (2006).

[4] David, L. *et al.* A high-resolution map of transcription in the yeast genome. *Proceedings of the National Academy of Sciences of the United States of America* **103**, 5320–5325 (2006).

[5] Xu, Z. *et al.* Bidirectional promoters generate pervasive transcription in yeast. *Nature* (2009).

[6] Steinmetz, L. M. *et al.* Dissecting the architecture of a quantitative trait locus in yeast. *Nature* **416**, 326–30 (2002).

[7] Wei, W. *et al.* Genome sequencing and comparative analysis of saccharomyces cerevisiae strain yjm789. *Proc Natl Acad Sci USA* **104**, 12825–30 (2007).

[8] Storey, J. & Tibshirani, R. Statistical significance for genomewide studies. *Proceedings of the National Academy of Sciences* **100**, 9440–9445 (2003).

[9] Kang, H. *et al.* Variance component model to account for sample structure in genome-wide association studies. *Nature genetics* **42**, 348–354 (2010).

[10] Segura, V. *et al.* An efficient multi-locus mixed-model approach for genome-wide association studies in structured populations. *Nature genetics* (2012).

[11] Brem, R. B., Yvert, G., Clinton, R. & Kruglyak, L. Genetic dissection of transcriptional regulation in budding yeast. *Science* **296**, 752–5 (2002).

[12] Yvert, G. *et al.* Trans-acting regulatory variation in saccharomyces cerevisiae and the role of transcription factors. *Nat Genet* **35**, 57–64 (2003).

[13] Pierce, S. E., Davis, R. W., Nislow, C. & Giaever, G. Genome-wide analysis of barcoded saccharomyces cerevisiae gene-deletion mutants in pooled cultures. *Nature Protocols* **2**, 2958–74 (2007).

[14] Steinmetz, L. M. *et al.* Systematic screen for human disease genes in yeast. *Nature Genetics* **31**, 400–4 (2002).

[15] Chen, L., Emmert-Streib, F. & Storey, J. Harnessing naturally randomized transcription to infer regulatory relationships among genes. *Genome Biology* **8**, R219 (2007).

[16] Schadt, E. E. *et al.* An integrative genomics approach to infer causal associations between gene expression and disease. *Nature genetics* **37**, 710–717 (2005).

[17] Chen, B.-J. *et al.* Harnessing gene expression to identify the genetic basis of drug resistance. *Molecular systems biology* **5** (2009).

[18] Storey, J. The positive false discovery rate: A bayesian interpretation and the q-value. *Annals of Statistics* 2013–2035 (2003).

[19] MacIsaac, K. D. *et al.* An improved map of conserved regulatory sites for Saccharomyces cerevisiae. *BMC Bioinformatics* **7**, 113 (2006).

[20] Hogan, D. J., Riordan, D. P., Gerber, A. P., Herschlag, D. & Brown, P. O. Diverse RNA-binding proteins interact with functionally related sets of RNAs, suggesting an extensive regulatory system. *PLoS Biology* **6**, e255 (2008).
